# *De novo* transcriptome assembly reveals high transcriptional complexity in *Pisum sativum* axillary buds and shows rapid changes in expression of diurnally regulated genes

**DOI:** 10.1186/s12864-017-3577-x

**Published:** 2017-03-02

**Authors:** Stephanie C. Kerr, Federico Gaiti, Christine A. Beveridge, Milos Tanurdzic

**Affiliations:** The University of Queensland, School of Biological Sciences, St Lucia, QLD 4072 Australia

**Keywords:** *Pisum sativum*, RNA-seq, Transcriptome, Gene expression, Long non-coding RNA, Axillary buds, Diurnal

## Abstract

**Background:**

The decision for a bud to grow into a branch is a key regulatory process affecting plant architecture. In order to study molecular processes regulating axillary bud outgrowth in the model plant garden pea (*Pisum sativum*), we sequenced the axillary bud transcriptome and performed *de novo* transcriptome assembly.

**Results:**

We assembled a pea axillary bud transcriptome into 81,774 transcripts comprised of 194,067 isoforms. This new pea transcriptome resource is both comprehensive and representative, as shown by comparison to other available pea sequence resources. Over half of the transcriptome could be annotated based on sequence homology to *Arabidopsis thaliana* proteins, while almost one quarter of the isoforms were identified as putative long non-coding RNAs (lncRNAs). This transcriptome will be useful in studies of pea buds because it includes genes expressed specifically in buds which are not represented in other transcriptome studies. We also investigated the impact of a short time collection series on gene expression. Differential gene expression analysis identified 142 transcripts changing within the short 170 min time frame that the buds were harvested within. Thirty-three of these transcripts are implicated in diurnal fluctuations in other flowering plants, while the remaining transcripts include 31 putative lncRNA. Further investigation of the differentially expressed transcripts found an enrichment of genes involved in post-transcriptional regulation, including RNA processing and modification, as well as genes involved in fatty acid biosynthesis and oxidative phosphorylation.

**Conclusions:**

We have sequenced and assembled a high quality pea bud transcriptome containing both coding and non-coding RNA transcripts that will be useful for further studies into axillary bud outgrowth. Over the short sample collection time frame of just 170 min, we identified differentially expressed coding and non-coding RNA, some of which are implicated in diurnal regulation, highlighting the utility of our transcriptome resource in identifying gene expression changes and informing future experimental designs.

**Electronic supplementary material:**

The online version of this article (doi:10.1186/s12864-017-3577-x) contains supplementary material, which is available to authorized users.

## Background

Branching is a major determinant of shoot architecture in plants and highly influences the yield of agricultural crops. The formation of branches begins when small meristematic tissue develops in the axils of leaves to form axillary buds [1]. Once formed these buds usually remain in a state of suspended growth (dormant) until they receive a growth-triggering signal. These signals may be environmental, such as light, nutrients, and decapitation of the shoot tip, or endogenous, such as hormones and sugars [2]. The ability of a plant to respond to each of these signals determines its final shoot architecture.

There is still much debate and poor clarity on exactly how axillary buds are maintained dormant or promoted to grow out [1, 2]. Multiple signaling pathways are known to regulate this process, but little is known about how these pathways are integrated and which signaling pathways predominate at which stages of development. For example, it has recently been shown that sugars are the likely initial trigger of bud outgrowth after decapitation of the shoot tip [3], but whether sugars also interact with hormone or other environmental signaling pathways is yet to be determined. Therefore, in order to better understand the genetic changes involved in bud outgrowth, we sequenced the first transcriptome of axillary buds from garden pea (*Pisum sativum*) plants. Garden peas have large buds separated by long internodes, making it easier to harvest bud tissue specifically. In addition, branching has been well studied in garden pea with many resources available, including branching mutants and curated transcriptome libraries [4].

This is the first of two gene expression studies of pea axillary buds using this new transcriptome resource. Here we report and describe the pea axillary bud transcriptome and identify a significant time-of-day experimental variable influencing gene expression during the sample collection time frame in the *rms5-3* genetic background. These mutants are near-isogenic with the wild type cv. Torsdag widely used in developmental genetics and hormone studies, and produce very little endogenous strigolactone, a plant hormone which represses axillary bud outgrowth [2] therefore enabling inclusion of transcripts involved in active bud outgrowth. In addition to establishing a quality pea bud transcriptome, we identified gene expression changes in the growing axillary buds samples at different time windows over a 170 min time frame that are contributed by diurnal and circadian effects. These results have implications for future design of gene expression studies as most gene expression studies that utilize multiple treatments and replicates require the samples to be harvested over a period of time.

## Results and discussion

### Transcriptome sequencing and de novo garden pea axillary bud transcriptome assembly

We generated a pea axillary bud *de novo* transcriptome assembly from ~55 million 250 bp paired-end RNA-seq reads sequenced using Illumina MiSeq technology (Table 1; Additional file 1: Tables S1 and S2). The transcriptome comprised of reads from both mock and strigolactone treated buds of intact *rms5-3* mutant plants treated for 1, 2, 4 and 6 h, with each treatment time harvested over a 170 min time frame (Additional file 1: Table S2). The assembly produced 81,774 transcripts with 194,067 isoforms, an N50 isoform size of 2170 bp (Table 1) and a transcriptome size of 57 Mb (Table 2). The isoform length distribution is shown in Additional file 1: Figure S1. The high number of assembled transcripts likely reflects the complex nature of a growing axillary bud.

**Table 1:** Summary of *de novo* pea axillary bud transcriptome statistics

Statistic	Pea bud transcriptome
# contigs	81,774
# isoforms	194,067
Shortest isoform (bp)	201
Longest isoform (bp)	17,155
# large isoforms (>1000 bp)	108,011
N50 isoforms (bp)	2170
Ave. isoform length (bp)	1285
# paired reads used in assembly	~55 million
# nucleotides used in assembly	~23 Gb
Reads mapped in pairs (%)	58.67%
Individual reads mapped (%)	90.94%

**Table 2:** Estimated size of different *Pisum sativum* and other plant species transcriptomes

Species	Source	Estimated transcriptome size (Mb)
*Pisum sativum* bud	-	57^a^
*Pisum sativum* bud	-	249^b^
*Pisum sativum*	[7]	37
*Pisum sativum*	[6]	10
*Pisum sativum*	[8]	59
*Pisum sativum* (Kaspa)	[5]	81^b^
*Pisum sativum* (Parafield)	[5]	72^b^
*Medicago truncatula*	Mt4.0v1	67^c^
*Solanum lycopersicum*	ITAG1	38^c^
*Arabidopsis thaliana*	TAIR10	66^c^

^a^reflects the transcriptome size estimated using the longest isoform per contig

^b^reflects the transcriptome size estimated using all isoforms

^c^based on whole genome reference

### Assessment of the transcriptome assembly

A number of criteria were used to determine the redundancy and quality of our pea bud transcriptome assembly, including comparisons to published transcriptomes from pea and other plant species, and alignment to available pea gene sequences.

#### The pea axillary bud transcriptome redundancy and comparison to other pea gene sequences

Firstly, we tested within-assembly redundancy by comparing the assembled isoforms to all other isoforms using BLASTN (1E^−03^) and a minimum hit coverage of 80% (Additional file 1: Table S3). Most of the matches were between isoforms from the same transcript, with only six matches between different transcripts. This suggests a very low redundancy of transcripts in our assembly.

As a number of pea transcriptomes have previously been published [5–8], we compared them with our pea bud transcriptome. Over 90% of the pea transcripts generated using earlier 454 sequencing technology [6–8] are contained within our transcriptome assembly (Table 3). In line with this, our transcriptome contained significantly more transcripts than the other transcriptomes (Table 3). These results are likely due to the increased sequence read length and depth used in our transcriptome sequencing, resulting in a more comprehensive transcriptome assembly and discovery of novel pea genes. This is further corroborated by comparisons to the more recent pea transcriptomes of the Kaspa and Parafield genotypes [5] that used similar Illumina sequencing technology to ours. In these cases, we found that ~75% of our isoforms are present in the Kaspa and Parafield transcriptomes, while ~78% of the Kaspa and Parafield transcripts were present in our transcriptome (Table 3). It is worth noting that all of the published pea transcriptomes comprise a number of different plant tissues while ours is restricted to the axillary bud. This may imply that many of the differences in overlap are the result of true biological differences between tissues, highlighting the complexity of the transcriptome of the developing axillary bud.

**Table 3:** BLAST comparisons (E-value 1E^−10^) between the pea bud isoforms as the query and *Pisum sativum* contigs [5–8] or *Medicago* CDS and proteins as the reference

Reference	Analysis	# reference matches	# pea bud isoform matches
Pea (Franssen) contigs	BLASTN	78,692 (93%)	109,619 (56%)
Pea (Kaur) contigs	BLASTN	13,112 (96%)	46,411 (24%)
Pea (Duarte) contigs	BLASTN	65,732 (96%)	112,429 (58%)
Pea (Kaspa) contigs	BLASTN	98,613 (79%)	143,092 (74%)
Pea (Parafield) contigs	BLASTN	112,836 (77%)	147,661 (76%)
*Medicago* (Mt4.0v1) CDS	TBLASTX	48,203 (77%)	117,699 (61%)
*Medicago* (Mt4.0v1) proteins	BLASTX	47,385 (76%)	115,151 (59%)

We also found that approximately 80% of pea protein sequences available in the Swiss-Prot and UniProtKB databases had a matching transcript in the transcriptome, using BLASTX (1E^−10^) with a minimum hit coverage of 80% (Table 4). Therefore, our comprehensive *de novo* transcripts assembly has allowed us to generate a representative catalog of genes expressed in a pea bud.

**Table 4:** BLASTX (E-value 1E^−10^) comparisons between the pea bud isoforms as the query and Ultra-Conserved Ortholog (UCO) protein sequences, Swiss-Prot pea or UniProtKB pea protein sequences as the reference

Reference	Min. subject coverage	# reference matches	# pea bud isoform matches
UCO	75%	316 (81%)	320
Swiss-Prot	80%	308 (79%)	812
UniProtKB	80%	1210 (80%)	2289

#### Inter-specific sequence comparison

In the absence of garden pea reference genome sequences, we used the coding sequences (CDS) annotated in the whole genome assembly of the closest available relative, *Medicago truncatula* (Mt4.0v1 [9]), to annotate the axillary bud transcriptome isoforms using TBLASTX (1E^−10^). Over 60% of the pea isoforms matched to a *Medicago* CDS, while ~77% of the *Medicago* CDS matched a pea isoform (Table 3). The discrepancy between the pairwise TBLASTX searches is likely explained by a combined effect of pea-specific and non-coding transcripts which is especially likely given the almost 9-fold increase in the garden pea genome size relative to *Medicago* [4], as well as alternatively spliced isoforms or potential mis-assemblies.

Furthermore, we compared our transcriptome to a list of 387 Ultra Conserved Orthologs (UCOs). These are single copy genes conserved across eukaryotes, specifically *Arabidopsis thaliana,* humans, mice, yeast, fruit flies and *Caenorhabditis elegans* [10]. Using BLASTX (1E^−10^) and a minimum 75% sequence coverage, we found 81% of the UCO sequences in our pea bud transcriptome (Table 4).

#### Comparison to single copy gene sequences from pea

We used an arbitrarily selected set of ten previously sequenced single copy pea genes [11–19] to determine how well each transcript has been assembled. BLASTX (1E^−100^) was used to compare these ten reference sequences against the pea bud transcriptome to determine how many copies of each gene was present in the transcriptome, and how well each transcript was assembled. Only two of the ten reference sequences, *PsFed-1* and *PsRMS5*, had more than one copy in the transcriptome (Additional file 1: Table S4). Both of these transcripts had low read coverage over the reference transcript which could have impacted on the ability to properly assemble full-length transcripts (Additional file 2: Figures S5 and S11). Importantly, six of the reference sequences are matched by a single isoform covering the majority of the reference sequence (Additional file 1: Table S4; Additional file 2: Figures S2-S11), reflecting the high level of completeness of our assemblies. In support of this, eight of the assembled transcripts match at their 5′ end with the 5′ end of the reference sequences (Additional file 1: Figures S2-S11). As expected, transcript abundance affects this analysis, such that the transcripts of three pea genes with low abundance, *PsRMS5*, *PsFed-1*, and *PsPETE*, are found only partially assembled in the pea axillary bud transcriptome.

### Annotation

In order to predict the putative function of the pea bud transcripts and their isoforms, BLASTX was used to align the isoforms, firstly with *Arabidopsis thaliana* proteins (1E^−10^), and secondly with all proteins from the NCBI non-redundant (nr) sequence database (1E^−10^). This led to 55 and 61% of the isoforms, and 27 and 34% of the transcripts being annotated by protein sequence similarity, respectively (Additional file 3: Table S5; Additional file 4: Table S6).

**Fig. 1:**
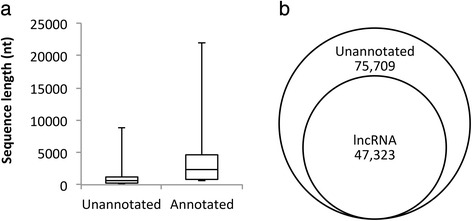
**a** Comparison of the sequence length (nt) of unannotated and annotated isoforms from the *de novo* pea axillary bud assembly, and **b** the overlap between the number of unannotated isoforms and the number of lncRNA in the pea bud transcriptome

When we compared the length of the isoforms annotated with the nr protein sequence database with the unannotated isoforms, we found that the average unannotated isoform was 472 bp in length while the average annotated isoform was ~4 times longer, with an average length of 1805 bp (Fig. 1a). To account for the fact that some of the isoforms may contain untranslated regions, we also determined the open-reading frame (ORF) sizes of the isoforms. The average ORF size was 65 bp for the unannotated isoforms and 320 bp for the annotated isoforms, a similar fold-change to the difference in overall length between the two types of isoforms. The unannotated isoforms are mostly quite short in length, which suggests either that they are incomplete assemblies and so cannot be matched adequately to their homologues in other species, or that they may be non-coding RNAs.

To identify putative long non-coding RNAs (lncRNAs) in the pea bud transcriptome, we used a custom lncRNA prediction computational pipeline which took into account four core filtering criteria: 1) similarities to known protein sequences and protein domains, 2) the presence of signal peptides, 3) isoform length (>200 nt), and 4) open reading frame size (ORF < 50 aa). Using this approach, we found 47,322 putative lncRNAs (Additional file 5: Table S7), with a median length of 293 nt and a range from 201 to 2781 nt (Additional file 1: Figure S12). Two-thirds of the unannotated isoforms were identified as putative lncRNAs (Fig. 1b); conversely, none of the annotated isoforms were putative lncRNAs.

The large number of putative lncRNAs in pea is at the higher end of the range of the number of lncRNAs predicted in other plants species with a genome sequence reference [20–22]. This could be the result of a much larger non-coding portion of the pea genome [23], and it would imply that some of the non-coding portion of the pea genome is transcribed. Indeed, sequence comparison between the predicted lncRNA and available pea repetitive DNA sequences [23] revealed that 12,034 (25%) of the predicted lncRNA represent various types of repetitive portions of the pea genome, with transcripts of the LTR retrotransposons Ogre and Maximus, as well as unclassified repeats, representing 68% of all repetitive DNA transcripts (Additional file 1: Table S8). Furthermore, in the absence of a reference pea genome sequence, isoforms of lncRNA are not easily identifiable, potentially leading to inflated lncRNA counts. In addition, our RNA purification did not include a polyA+ selection step; therefore it is likely that some of the lncRNA may represent non-polyadenylated transcripts. Recently, novel non-polyadenylated transcripts have been detected in the model plants *Arabidopsis* and rice [24, 25]. These non-polyadenylated transcripts, known as intermediate sized ncRNAs (im-ncRNAs), are 50–300 nt in length, have low protein-coding potential, and do not show sequence similarity to any known ncRNA [24, 25].

Sequence comparisons to lncRNAs from *Medicago* [21] and *Arabidopsis* [20] were also made, identifying 1485 (6%) and 114 (0.3%) lncRNAs, respectively, conserved in pea (Additional file 5: Tables S9 and S10). As lncRNA are thought to be fast evolving and constrained by their secondary and tertiary structures [26, 27] and sometimes synteny [28], rather than just their sequence, detecting hundreds of sequence-conserved lncRNA provides a new resource for comparative analysis of lncRNA sequence and structure conservation in flowering plants.

**Fig. 2:**
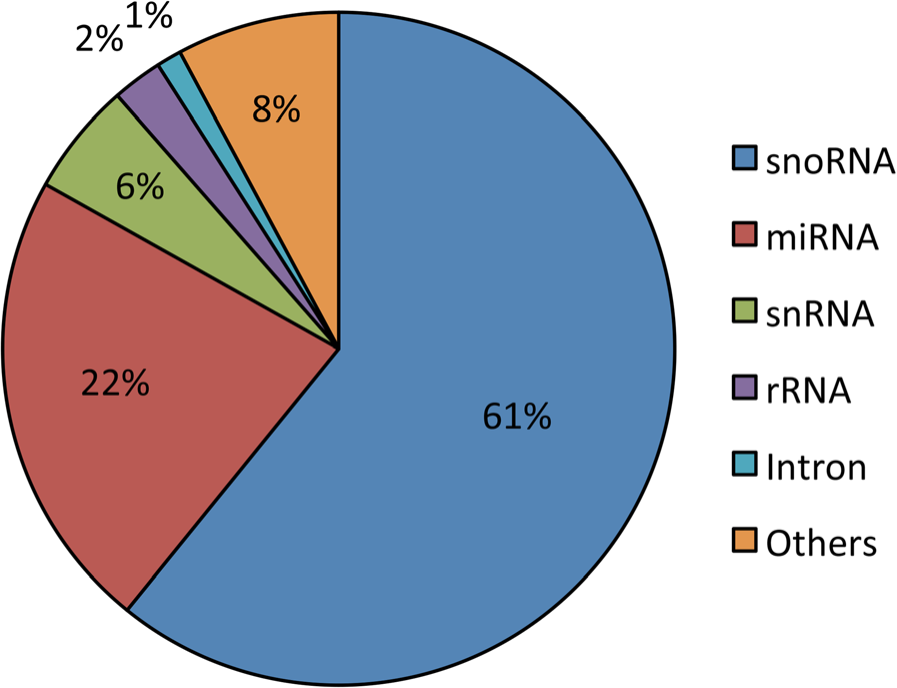
Rfam non-coding RNA families represented in the pea bud transcriptome

The pea bud transcriptome was also annotated with the Rfam database of RNA families [29]. A total of 921 isoforms and 354 transcripts were annotated in this way (Additional file 5: Table S11), of which small nucleolar RNAs (snoRNAs) and microRNA (miRNA) precursors (Fig. 2) were the most highly represented RNA families. Interestingly, we identified 100 likely miRNA precursor transcripts representing 31 miRNA gene families expressed in the pea axillary bud (Additional file 5: Table S11).

### Functional annotation of the pea axillary bud transcriptome

**Fig. 3:**
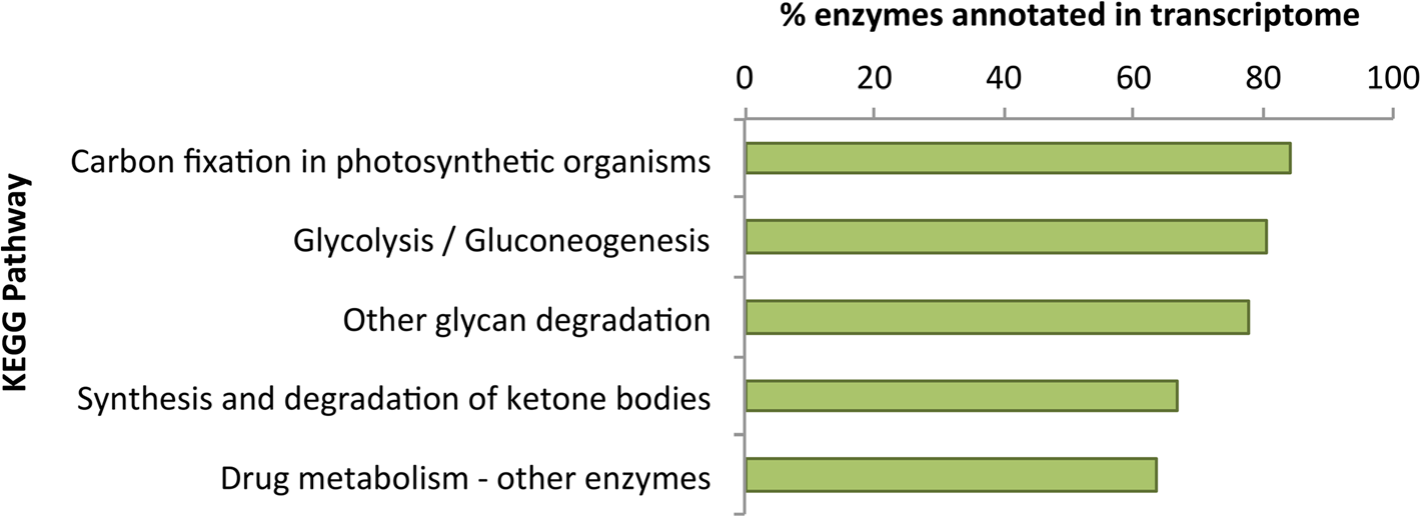
Enzyme representation of the top five KEGG pathways ranked by the percentage of enzymes annotated in the pea axillary bud transcriptome

We functionally annotated the pea axillary bud transcriptome using both gene ontology (GO) terms [30] and KEGG metabolic pathways [31]. We identified 140 KEGG pathways with at least one member of the pathway annotated in the pea bud transcriptome, and at least 20 KEGG pathways with more than 50% represented in the pea bud transcriptome (Additional file 5: Table S12). The KEGG pathways with the highest percentage of enzymes annotated in the transcriptome were carbon fixation in photosynthetic organisms with 21/25 (82%) enzymes annotated in the transcriptome, and glycolysis/gluconeogenesis with 25/31 (80%) of the enzymes annotated in the transcriptome (Fig. 3; Additional file 1: Figure S13). This, along with the most highly represented GO categories which included numerous metabolic processes (Additional file 5: Tables S13-S15), supports the fact that active and diverse metabolic processes are occurring in the photosynthetically active buds as they are growing.

### Diurnally regulated transcripts in pea axillary buds

To determine whether time of day had an effect on gene expression in axillary buds over the 170 min time frame of the collection of the samples, we used the RNA-seq data from our mock treated samples for differential gene expression analysis contrasting gene expression between the three collection time windows. In addition to identifying diurnally regulated genes in pea buds (based on expression and annotation), this analysis will inform future experimental design on axillary bud growth dynamics and gene expression studies. As the samples were collected in three groups between 1.30 and 4.30 pm (Additional file 1: Table S16), we designated samples harvested between 1.30 and 2.10 pm as time window 1, samples harvested between 2.10 and 2.50 pm as time window 2, and samples harvested between 3.50 and 4.30 pm as time window 3 as described in the Methods section.

**Fig. 4:**
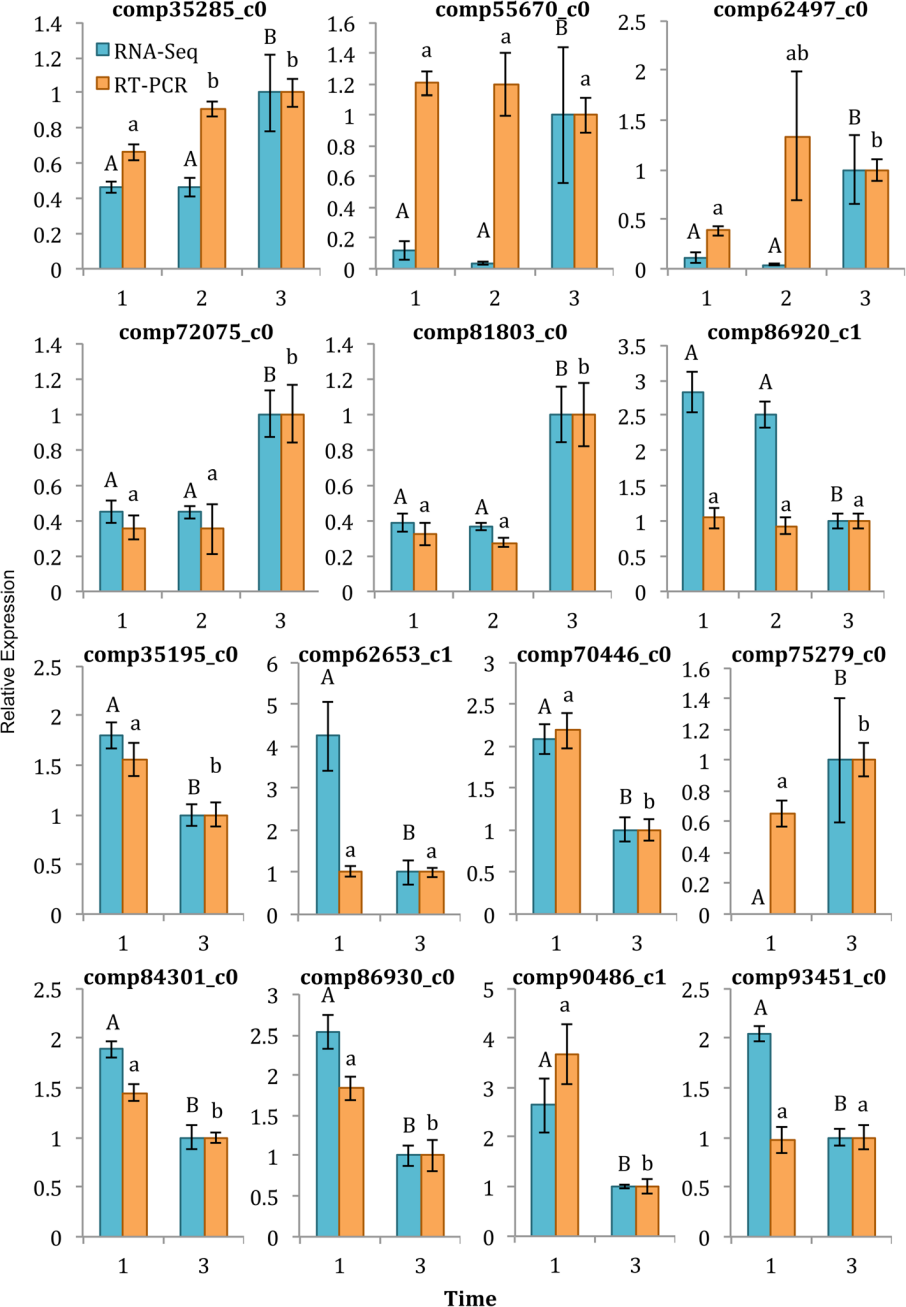
Validation of 14 differentially expressed transcripts using qRT-PCR. Transcript expression in node 2 *rms5-3* axillary buds was calculated relative to time window 3 for both RNA-seq data (*blue bars*) and qRT-PCR data (*orange bars*). Only time windows that were statistically significantly different in the RNA-seq edgeR analysis were included. Data are means ± SE (*n* = 3–4 pools of ~60 plants). Statistically significant differences were determined using a one-way ANOVA with a post-hoc Tukey’s test. Different letters (*capital letters* for RNA-seq values, *lower case letters* for qRT-PCR values) represent statistically significant differences at FDR<0.05 or *p* < 0.05, respectively

Pairwise comparisons of gene expression differences were made between each of the time windows (Table 5; Additional file 1: Tables S17 and S18), which identified a total of 142 unique differentially expressed (DE) transcripts. The time windows harvested closer together showed fewer DE transcripts between them, with no DE transcripts between time windows 1 and 2, and only 37 DE transcripts between time windows 2 and 3. The time windows that were the furthest apart, time windows 1 and 3, showed 124 DE transcripts. There was an overlap of 19 DE transcripts between the two analyses. We tested 14 randomly chosen DE transcripts by qRT-PCR; all 14 were identified as DE in our differential gene expression analysis between time windows 1 and 3, while six were identified as DE in the analysis between time windows 2 and 3. The qRT-PCR results showed ten out of the 14 (71%) transcripts were validated between time windows 1 and 3, while only two out of six (33%) were validated between time windows 2 and 3 (Fig. 4). For the genes that were validated by qRT-PCR, very similar fold changes were identified by both the edgeR analysis and qRT-PCR (e.g., see comp 72075_c0 and comp 81803_c0 in Fig. 4). Notably, due to the short time differences, these fold-changes were generally less than 3-fold.

**Table 5:** Differentially expressed transcripts (FDR < 0.05) between pairwise comparisons of each time window collected over a time frame of 170 min in *Pisum sativum* buds

Pairwise comparison	Down	Up	Total	Annotated	Potential long non-coding RNA
Time window 1 vs 2	0	0	0	n/a	n/a
Time window 1 vs 3	58	66	124	62 (50%)	28 (23%)
Time window 2 vs 3	5	32	37	13 (35%)	5 (14%)
Unique transcripts^a^	61	81	142	71 (50%)	31 (22%)

^a^represents unique transcripts from all three pairwise comparisons

Only half of the total unique DE transcripts were annotated (Tables 5 and 6), which suggested an involvement of pea-specific genes, including non-coding RNA. This was confirmed as 44% of the unannotated DE transcripts were classified as putative lncRNA using our criteria (Table 5; Additional file 1: Table S19); however, none of the DE transcripts showed sequence homology to Rfam and repetitive DNA databases. This suggests that as well as affecting expression of protein-coding genes, the time of sample collection also affects expression of putative lncRNAs. As not all of the unannotated transcripts were classified as putative lncRNA, the remaining unannotated transcripts could have other functions such as encoding as yet unknown or pea-specific proteins or small RNA precursors.

**Table 6:** List of annotated differentially expressed transcripts identified in pairwise comparisons (FDR < 0.05) between the three time windows in *Pisum sativum* buds collected over a 170 min time frame

Transcript	AT#	*Arabidopsis* protein annotation
comp103811_c0	AT5G16080	Probable carboxylesterase 15
comp34724_c0	AT4G30110	Cadmium/zinc-transporting ATPase HMA2
comp34931_c0	AT1G15760	Sterile alpha motif domain-containing protein
comp35134_c1	AT5G02570	Histone h2b
comp35188_c0	AT2G43460	60s ribosomal protein l38
comp35195_c0	AT3G62980	Protein TRANSPORT INHIBITOR RESPONSE 1
comp35285_c0	AT1G14450	NADH dehydrogenase
comp35297_c0	AT4G26470	Calcium-binding protein CML21
comp35867_c0	AT2G16365	F-box protein
comp36828_c0	AT3G48770	ATP/DNA binding protein
comp42921_c0	AT1G68050	Kelch repeat-containing protein
comp54867_c0	AT1G02070	Uncharacterized protein
comp55051_c0	AT1G27480	Lecithin-cholesterol acyltransferase-like 1
comp55149_c0	AT3G24150	Uncharacterized protein
comp55670_c2	ATMG00030	Uncharacterized mitochondrial protein ATMG00030
comp55866_c0	AT4G00100	40s ribosomal protein s13-2
comp55974_c0	AT3G61610	Galactose mutarotase-like superfamily protein
comp64053_c1	AT3G12587	Oligosaccaryltransferase
comp68925_c0	AT3G55340	Phragmoplastin interacting protein 1
comp69006_c0	AT3G47570	Receptor kinase
comp70446_c0	AT5G24930	Zinc finger protein constans-like 4
comp70806_c0	AT1G75540	Constans-like b-box zinc finger protein
comp71289_c1	AT1G07770	40s ribosomal protein s15a
comp71932_c0	AT5G25450	Cytochrome bd ubiquinol oxidase
comp72075_c0	AT1G04400	Cryptochrome 2
comp73339_c0	AT3G11050	Ferritin 2
comp75279_c0	AT2G03340	WRKY transcription factor 3
comp75525_c2	AT2G37620	Actin 1
comp77858_c0	AT4G40030	Histone h3
comp77929_c0	AT4G29390	40s ribosomal protein s30
comp78122_c1	AT3G54500	Night light-inducible and clock-regulated 2
comp78315_c0	AT1G78510	Solanesyl diphosphate synthase 1
comp79509_c0	AT1G69180	Transcription factor crc
comp79848_c1	AT5G24780	Acid phosphatase VSP1
comp80157_c2	AT1G07050	CCT motif family protein
comp81803_c0	AT5G42900	Cold regulated protein 27
comp82468_c1	AT2G05960	Retroelement pol polyprotein
comp82517_c0	AT5G02560	Histone h2a
comp83232_c1	AT3G15620	UV repair defective 3
comp83562_c0	AT4G38960	B-box type zinc finger-containing protein
comp83593_c0	AT2G25530	AFG1-like ATPase family protein
comp83707_c0	AT3G49430	Ser arg-rich protein 34a
comp84080_c0	AT5G35970	P-loop containing nucleoside triphosphate hydrolases superfamily protein
comp84301_c1	AT2G24820	Translocon at the inner envelope membrane of chloroplasts 55
comp84585_c0	AT5G58140	Phototropin 2
comp85037_c0	AT4G24290	Mac perforin domain-containing protein
comp85451_c1	AT2G44740	Preg1-like negative regulator
comp85721_c1	AT2G42750	DNAJ heat shock N-terminal domain-containing protein
comp86930_c0	AT2G40130	Double clp-n motif-containing p-loop nucleoside triphosphate hydrolases superfamily protein
comp87035_c5	AT2G38540	Nonspecific lipid-transfer protein
comp87071_c2	AT3G61130	Galacturonosyltransferase 3
comp87121_c0	AT5G66290	Uncharacterized protein
comp87227_c2	AT1G18330	MYB-related transcription factor EPR1
comp87716_c1	AT3G62330	CCHC-type zinc knuckle protein
comp88021_c0	AT1G56220	Dormancy auxin associated protein
comp89415_c1	AT3G20810	Jumonji-C domain-containing protein 30
comp89617_c0	AT5G24850	Cryptochrome 3
comp89772_c0	AT5G56850	Uncharacterized protein
comp90486_c1	AT5G24470	Pseudo-response regulator 5
comp90925_c6	AT3G19900	Uncharacterized protein
comp91531_c2	AT3G14050	RelA-SpoT like protein RSH2
comp91844_c1	-	Hypothetical protein
comp91844_c4	-	Hypothetical protein
comp92821_c0	AT4G16146	cAMP-regulated phosphoprotein 19-related protein
comp93257_c0	AT3G20390	Endoribonuclease L-PSP family protein
comp93445_c0	AT5G04800	40s ribosomal protein S17-4
comp93451_c0	AT1G04220	Beta-ketoacyl-synthase
comp93952_c0	AT3G55280	60s ribosomal protein L23a-2
comp94434_c0	AT3G06730	M-type thioredoxin
comp95443_c0	AT5G08180	Ribosomal protein L7ae-like
comp99868_c0	AT4G29040	Regulatory particle AAA-ATPase 2A

lncRNAs have previously been associated with diurnal changes and light responses [32]. Hazen et al. [33] looked for non-coding sequences that exhibited rhythmic expression and identified 1052 intergenic regions of the *Arabidopsis* genome that had rhythmic expression. In addition, 7% of protein-coding genes exhibited rhythmic expression of lncRNA on the antisense strand, otherwise known as natural antisense transcripts (NATs). Interestingly, they also found that a number of circadian clock genes had NATs that exhibited diurnal fluctuations. This included *PSEUDORESPONSE REGULATOR* (*PRR5*), a transcriptional repressor that regulates key clock genes [34], which was also identified as DE in our study; the transcript comp90486_c1 assembly contained six isoforms, five of which were annotated as PRR5 and one of which was annotated as a putative lncRNA. Unfortunately, as our libraries are not stranded, we were unable to confirm the putative lncRNA isoform as a *bona fide* NAT. We also found that ten of the DE transcripts identified in our analysis were identified by Nakamichi et al. [34] as being bound and/or upregulated by PRR5 (Additional file 1: Table S20).

### Enrichment analysis

To examine the function of the DE transcripts, we performed a Fisher’s Exact Test (FDR < 0.05) to identify GO terms that were enriched in the annotated DE transcript data set compared to the reference transcriptome.

**Fig. 5:**
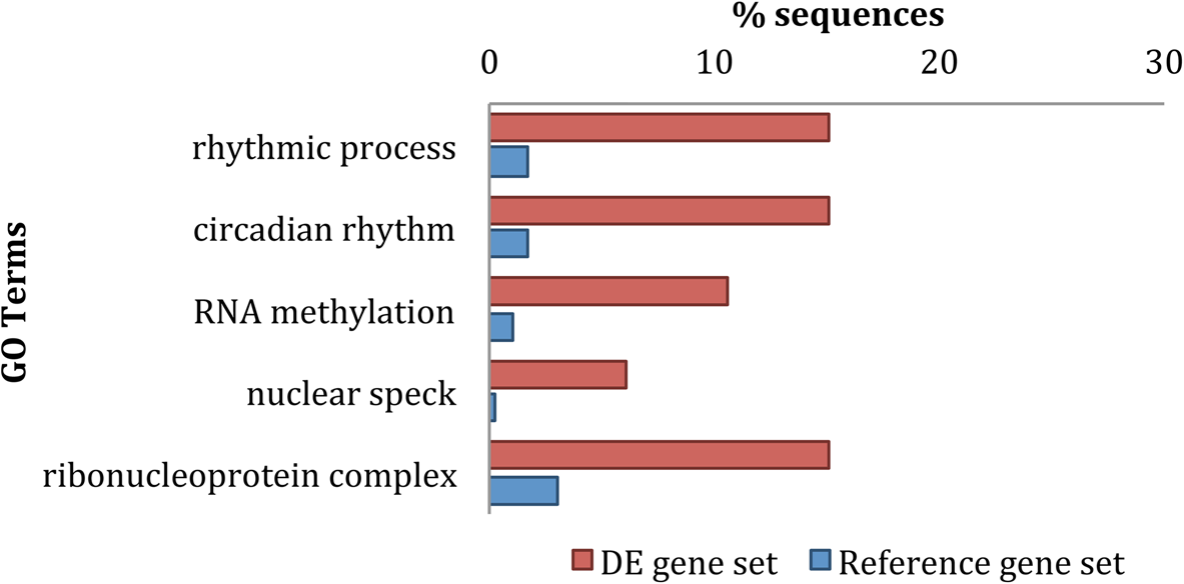
GO terms enriched in the differential expression analysis according to Fisher’s Exact Test (FDR < 0.05), and ordered according to % of sequences annotated in the DE gene set (*red*) and the reference gene set (*blue*). The longest isoform for each transcript in the transcriptome was used in the analysis

There was a significant, 9-fold enrichment in the number of DE transcripts that were annotated with the GO terms rhythmic process and circadian rhythm (Fig. 5), which reflects the diurnal changes occurring in the buds. We have identified 13 genes in this set that have been characterized in the literature as being light-regulated or circadian clock-associated (Additional file 1: Table S21). In addition, 29 (40% of annotated transcripts) DE genes identified in this study were previously identified as diurnally regulated by Blasing et al. [35] (Additional file 1: Table S22).

Importantly, we also identified six (40%) GO terms related to post-transcriptional modifications including RNA methylation, ribonucleoprotein complex and nuclear speck (Fig. 5). This finding is consistent with previous studies showing a role of post-transcriptional regulation in diurnal gene expression changes [32, 36].

**Fig. 6:**
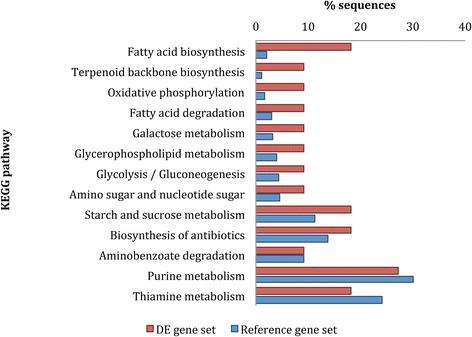
KEGG pathways represented in the differential expression analysis, ordered according to fold change difference between the percentage of genes annotated in the DE gene set (*red*) and the reference gene set (*blue*). The longest isoform for each transcript in the transcriptome was used in the analysis

We also examined the KEGG pathways that were represented by the DE genes by comparing the percentage of annotated DE genes annotated in the each KEGG pathway with the percentage of annotated reference genes present in each KEGG pathway. We found that genes involved in the fatty acid biosynthesis pathway had the largest increase in the DE gene set, with a 9-fold change (Fig. 6). Other large increases occurred in the oxidative phosphorylation pathway (5-fold; Fig. 6).

3-KETOACYL SYNTHASE 2 (KAS2; comp93451_c0), which is involved in the first steps of very-long-chain fatty acid biosynthesis [37], and LONG-CHAIN ACYL-COA SYNTHETASE 1 (LACS1; comp87716_c1), which preferentially modifies very long chain fatty acids (VLCFAs) for wax synthesis and long-chain fatty acids for cutin synthesis [38], were both identified as DE in this study. Fatty acid synthesis occurs in the plastid to provide the components of cell membranes for all plant cells [39], and has previously been linked to diurnal changes [40, 41] and is also required for growth.

Increased requirements for energy needed for bud growth are met by increases in ATP levels. Oxidative phosphorylation is a key stage of respiration that occurs in the mitochondria to synthesize ATP [42]. In agreement with this, enzymes from the mitochondria respiration complex I, NADH HYDROGENASE, (comp35285_c0), and complex III, UBIQUINOL CYTOCHROME C REDUCTASE SUBUNIT 7 (QCR7; comp71932_c0), were identified as DE in this study, and could potentially be regulated in a circadian fashion, likely to regulate the balance between glycolysis, oxidative phosphorylation and photophosphorylation, as suggested by Wagner et al. [43].

## Conclusions

We have presented a comprehensive pea bud transcriptome for which coding genes, putative lncRNAs, and miRNA precursors were identified. The assembly was validated by comparisons to other transcriptomes, which identified transcripts specific to pea axillary buds. Further analysis of this transcriptome found that growing pea buds contain many transcripts related to metabolic pathways, suggesting that the growing buds are highly active.

Gene expression was characterized in these growing axillary buds sampled in three time windows over a 170 min time frame. This analysis identified a number of transcripts changing over the short period of time and many of these could be annotated as genes with known or predicted diurnal regulation. It implies that changes in expression of genes known to be diurnally regulated are occurring quite rapidly in growing pea buds. The fact that we were able to discover a suite of differentially expressed genes over 170 min, and that some of them were annotated as diurnally related, indicates that our pea bud system should be sensitive enough to identify genes that are differentially expressed in response to experimental treatments. It also implies the need for consideration of fast diurnal changes in gene expression when designing gene expression studies in pea axillary buds. Further analysis discovered that a large proportion of the DE transcripts were putative lncRNAs and coding transcripts associated with post-transcriptional modifications.

## Methods

### Plant growth and harvest

*rms5-3* seeds were planted 4 per 2L pot containing potting mix (Green Fingers B2 Potting Mix; www.greenfingerspottingmix.com). *The rms5-3* (BL298) line used in this study was obtained after an initial cross between Wt15241 (rms5-3) and Torsdag (L107) (described in [44]), which was further backcrossed to Torsdag five times. Seedlings were grown in a randomized configuration until ~2.5 LE, or 7-days old, under 18-h day-length glasshouse conditions as described in [45]. Node 2 buds were treated with 10 μL of aqueous solution containing 0.1% Tween-20, 1% PEG 1450, 6.25% EtOH and either 0 or 1 μM of the synthetic strigolactone, *rac-*GR24, in acetone. Node 2 buds were harvested 1, 2, 4, or 6 h following treatment and immediately placed in liquid nitrogen. For the transcriptome sequencing all 24 samples were used, while for the DE analysis only the samples without the *rac*-GR24 treatment were used (12 odd-numbered samples 1–23 in Table S16). Approximately 30–40 buds from individual plants were pooled together to make one biological replicate, with four replicates collected for each of the three treatment windows. The ~840 buds were harvested between 13:30 and 16:20 (Additional file 1: Tables S2 and S16) on the 11th October 2012 at GPS coordinates (DMS) 27°29′43″ S, 153°0′36″ E. Those samples harvested between 1.30 and 2.10 pm were designated as time window 1, samples harvested between 2.10 and 2.50 pm as time window 2, and samples harvested between 3.50 and 4.30 pm as time window 3 (Table S16).

### RNA extraction, library construction and sequencing

RNA was extracted from samples using a TRIzol extraction method and then purified using RNeasy® MinElute® cleanup kit (Qiagen®). RiboZero^TM^ Magnetic (Plant leaf) kit (Epicentre®) was used to remove rRNA from 3.81 μg of each of the samples. The removal of rRNA was confirmed using a 2100 BioAnalyser (Agilent Technologies).

The RNA libraries were prepared using the ScriptSeq^TM^ V2 RNA Seq Library Preparation kit (Epicentre®), except for the following changes: the samples were incubated in the first step for 5 min at the lower temperature of 70 °C to reduce RNA fragmentation, the cDNA was purified using the MinElute® kit (Qiagen®), and the ScriptSeq^TM^ Index primers 1, 8, 9, 10, 11 and 12 (Epicentre®) were used as adaptors. Strand-specificity was not utilized. Size selection was performed by running the purified libraries on an agarose gel and excising a band of RNA between 350 and 550 bp long. This was then purified using the QIAquick® Gel Extraction kit (Qiagen®). The Qubit^TM^ dsDNA HS Assay was used to quantify the cDNA in each library.

Four libraries were pooled together for each sequencing run, with each library contributing 1.75 ng of RNA. The pooled libraries were prepared for sequencing using a MiSeq Desktop Sequencer (Illumina®) and run individually on a 150 or 250 paired-end cycle cartridge.

### RNA-seq read quality control

For each of the steps in this section, the R1 and R2 files were processed separately. FASTQC (http://www.bioinformatics.babraham.ac.uk/projects/fastqc/) was used on the reads produced by the Illumina® MiSeq to assess the quality. FASTX-Toolkit version 0.0.13.2 (http://hannonlab.cshl.edu/fastx_toolkit/index.html) was used for cleaning of the reads. FASTx trimmer was used to remove 10 bases from the 5′ ends of the reads and x bases from the 3′ ends, where x was determined for each sample individually depending on the FASTQC results. FASTx quality trimmer was used to remove bases from the ends of reads that had a Q-score lower than 20; any reads less than 15 bp long were discarded. FASTx quality filter was used to remove reads that had more than 50% of its bases with a Q-score less than 20. FASTx clipper was used to remove any adaptor sequences from the reads; any reads less than 5 bp long were discarded. Deconseq (version 0.4.2) was run locally to remove rRNA sequences from all of the samples [46]. Finally, any unpaired reads from the R1 and R2 files were removed.

### De novo transcriptome assembly

Samples 5, 8, 11, 20 and 21 were excluded from the *de novo* assembly due to low quality reads. The paired-end reads from the remaining samples were used in the assembly. Trinity software (version 2013-02-25) was used to assembly a *de novo* transcriptome from the paired-end reads with the default parameters, except a minimum k-mer coverage of 2 was specified [47, 48].

### Assessment of assembly

#### The pea axillary bud transcriptome redundancy and comparison to other pea gene sequences

BLASTN (version 2.2.28+) [49] was run locally to align the isoforms in the pea bud transcriptome to each other, specifying an E-value of 1E^−03^ and a hit coverage of 80%.

#### Comparing transcriptomes

BLASTN, BLASTX and TBLASTX (version 2.2.28+) [49] were run locally to align the sequences in each transcriptome (Additional file 1: Table S23) to the pea bud transcriptome, specifying an E-value of 1E^−10^.

#### Swiss-Prot and UniProtKB

BLASTX (version 2.2.28+) [49] was run locally to align UniProtKB and SwissProt databases (Additional file 1: Table S23) of pea proteins to the pea bud transcriptome, specifying an E-value of 1E^−10^ and a hit coverage of 80%.

#### Ultra-conserved orthologs (UCOs)

BLASTX (version 2.2.28+) [49] was used to compare the 357 UCO protein sequences (Additional file 1: Table S23) with the pea bud transcriptome, specifying an E-value of 1E^−10^ and a hit coverage of 75%.

#### Comparison to single copy sequenced genes in pea

BLASTN (version 2.2.28+) [49] was run locally to align the single copy pea genes to the transcriptome, specifying an E-value of 1E^−100^. The single copy pea genes were *PsApxI* [17], *PsBRC1* [11], *PsEXGT1* [19], *PsFed-1* [13], *PsHMG-1* [14], *PsKO1* [12], *PsPCNA* [18], *PsPETE* [16], *PsRMS4* [15], and *PsRMS5* [15].

### Gene annotation and ontology

To annotate the transcriptome, BLASTX (version 2.2.28+) [49] was run locally to align each isoform in the transcriptome to the nr database of *Arabidopsis thaliana* proteins and the whole nr database, specifying an E-value of 1E^−10^.

The GO and KEGG annotations were assigned by loading the *Arabidopsis thaliana* annotation into BLAST2GO [50] and using the mapping function to map the annotated genes.

A custom computational pipeline was used to predict long non-coding RNAs (lncRNAs). All isoforms were subjected to BLASTP 2.2.28+ (NCBI nr), BLASTX 2.2.28+ (NCBI nr), HMMER 3.0 (both Pfam-A and Pfam-B) [51], and SignalP 4.1 [52] searches. For BLASTP, HMMER, and SignalP analyses, the isoforms were translated (start to stop codon) by Getorf tool [53] and the longest unique ORF for each isoform was retained. Isoforms with an E-value less than 1E^−04^ in any of the search algorithms were considered protein-coding (for SignalP D-cutoff value of 0.45 was used). To reduce the number of potential spurious lncRNAs in the transcriptome, isoforms shorter than 200 nt were removed. Any remaining isoforms of uncertain coding potential were removed by applying a strict ORF size cut-off of 50 amino acids. Finally, the Coding Potential Calculator (CPC) [54] was used to evaluate the sensitivity of our computational pipeline. Only isoforms that were classified as ‘noncoding’ by CPC were finally classified as putative pea bud lncRNAs. The lncRNA isoforms were compared to a collection of annotated *Pisum sativum* repetitive DNA sequences from Macas et al. [23] using BLAST (1E^−10^).

Infernal (version 1.1) [29] was run locally to annotate the transcriptome with Rfam, specifying an E-value of 1E^−02^.

### Identification of differentially expressed transcripts

Read counts for Read 1 of all mock treated samples were calculated using the default parameters for RSEM (version 2013-04-12) [55], except that the reference file was produced using no-polyA. Differential expression of transcripts between the mock treated samples at time windows 1, 2 and 3 (Additional file 1: Table S16) was calculated using edgeR (Bioconductor version 3.2.4) [56]. The count tables were first filtered to remove any transcripts with less than ten read counts in total, and the library sizes were normalised. Dispersions were estimated using the Cox-Reid profile-adjusted likelihood method, and the matrix was fit to a generalized linear model (GLM). Pairwise comparisons were then made between time windows 1, 2 and 3, with three samples within each time window treated as biological replicates. To determine differentially expressed genes, an FDR threshold of 0.05 was used.

### qRT-PCR validation

RNA used for qRT-PCR validation was the same as was used for RNA sequencing. cDNA was synthesized from 500 ng RNA using the iScript^TM^ reverse transcription supermix (BioRad) as per the manufacturer’s instructions. The cDNA was diluted to 0.25 ng/μL for qRT-PCR.

qRT-PCR analyses were performed and analyzed as previously described [3]. Primer sequences were designed using Primer3 software [57] based on transcript sequences from the *de novo* transcriptome assembly and can be found in Additional file 1: Table S24. *PsTUBULIN2* was used as the reference gene.

### Enrichment analysis

BLAST2GO (version 3.0.9) [50] was used to determine enriched GO terms using a two-sided Fisher’s enrichment analysis with an FDR threshold of 0.05, and the longest isoform for each transcript was used as the reference dataset.

## Additional files

Additional file 1: Table S1. Summary of the statistics for the raw and cleaned MiSeq paired-end reads. **Table S2.** Read quality statistics for Read 1 and Read 2 of each of the 24 RNA libraries sequenced. **Figure S1.** Length distribution of the isoforms in the pea axillary bud transcriptome. **Table S3.** Comparison of the *Pisum sativum* bud transcriptome isoforms using BLASTN (1E^−03^) and specifying a minimum hit coverage of 80%. **Table S4.** BLASTX (1E^−100^) results comparing single copy pea reference sequences against the pea axillary bud transcriptome. **Figure S12.** Length distribution of the long non-coding RNA in the pea axillary bud transcriptome. **Table S8.** Summary of the number of sequences with similarity to a pea repetitive DNA sequence [23] using BLAST (1E^−10^). **Figure S13.** The carbon fixation in photosynthetic organisms KEGG pathway with enzymes highlighted that were identified in the pea axillary bud transcriptome. **Table S16.** Timetable of sample harvest for the differential expression analysis. **Table S17.** List of 37 transcripts identified as DE in a pairwise comparison between time window 2 and time window 3. **Table S18.** List of 124 transcripts identified as DE in a pairwise comparison between time window 1 and time window 3. **Table S19.** List of 31 DE transcripts annotated with a long non-coding RNA. **Table S20.** List of DE transcripts that are bound or upregulated by PRR5 [34]. **Table S21.** List of DE transcripts that have previously been identified as circadian clock-associated and/or light/diurnally-regulated [58–71]. **Table S22.** List of 29 DE transcripts identified as diurnally regulated in Blasing et al. [35]. **Table S23.** List of online resources used in this article and their sources. **Table S24.** List of primers used for qRT-PCR analysis. (PDF 253 kb)
Additional file 2: Figure S2. The number of reads aligning to the *PsApxl* mRNA sequence and the alignment between *PsApxl* mRNA and its best hit to the pea bud transcriptome, comp92406_c0_seq1. **Figure S3.** The number of reads aligning to the *PsBRC1* CDS and the alignment between *PsBRC1* CDS and its best hit to the pea bud transcriptome, comp78442_c0_seq1. **Figure S4.** The number of reads aligning to the *PsEXGT1* mRNA and the alignment between *PsEXGT1* mRNA and its best hits to the pea bud transcriptome, comp88216_c0_seq1, 5, 7, 8, 11, 13, 14. **Figure S5.** The number of reads aligning to the *PsFed-1* CDS and the alignment between *PsFed-1* CDS and its best hits to the pea bud transcriptome, comp55599_c1_seq1 and comp351273_c0_seq1. **Figure S6.** The number of reads aligning to the *PsHMG*-*1* mRNA and the alignment between *PsHMG*-*1* mRNA and its best hit to the pea bud transcriptome, comp81333_c0_seq1. **Figure S7** The number of reads aligning to the *PsKO1* mRNA and the alignment between *PsKO1* mRNA and its best hits to the pea bud transcriptome, comp71699_c1_seq1, 2, 3, 4. **Figure S8.** The number of reads aligning to the *PsPCNA* mRNA and the alignment between *PsPCNA* mRNA and its best hit to the pea bud transcriptome, comp93178_c0_seq1. **Figure S9.** The number of reads aligning to the *PsPETE* gene and the alignment between *PsPETE* gene and its best hit to the pea bud transcriptome, comp92360_c0_seq1. **Figure S10.** The number of reads aligning to the *PsRMS4* CDS and the alignment between *PsRMS4* CDS and its best hit to the pea bud transcriptome, comp97254_c0_seq1. **Figure S11.** The number of reads aligning to the *PsRMS5* CDS and the alignment between *PsRMS5* CDS and its best hits to the pea bud transcriptome, comp74555_c0_seq1 and comp27382_c0_seq1. (PDF 399 kb)
Additional file 3: Table S5. BLAST2GO annotation of the pea axillary bud transcriptome against *Arabidopsis thaliana* protein sequences. (XLSX 16823 kb)
Additional file 4: Table S6. BLAST2GO annotation of the pea axillary bud transcriptome against the non-redundant (nr) protein database. (XLSX 15158 kb)
Additional file 5: Table S7. List of putative long non-coding RNA in the pea axillary bud isoform transcriptome and their open reading frame (ORF) size. **Table S9.** List of putative long non-coding RNA in the pea axillary bud isoform transcriptome that match to lncRNA in *Medicago* [21]. **Table S10.** List of putative long non-coding RNA in the pea axillary bud isoform transcriptome that match to NAT lncRNA in *Arabidopsis* [20]. **Table S11.** Rfam hits for pea axillary bud isoforms. **Table S12.** KEGG Pathway annotation of the pea axillary bud transcriptome. **Table S13.** Biological processes GO annotation of the pea axillary bud transcriptome. **Table S14.** Cellular component GO annotation of the pea axillary bud transcriptome. **Table S15.** Molecular function GO annotation of the pea axillary bud transcriptome. (XLSX 4242 kb)

## Abbreviations

aa: Amino acid
ANOVA: Analysis of variance
ATP: Adenosine triphosphate
BLAST: Blast local alignment search tool
bp: Base pair
cDNA: Complementary deoxyribonucleic acid
CDS: Coding sequence
CPC: Coding potential calculator
cv: Cultivar
DE: Differentially expressed
DNA: Deoxyribonucleic acid
EtOH: Ethanol
E-value: Expect value
FDR: False discovery rate
Gb: Giga base pair
GLM: Generalized linear model
GO: Gene ontology
im-ncRNA: Intermediate-sized non-coding ribonucleic acid
KEGG: Kyoto encyclopedia of genes and genomes
lncRNA: Long non-coding ribonucleic acid
LTR: Long terminal repeat
Mb: Mega base pair
miRNA: Micro ribonucleic acid
mRNA: Messenger ribonucleic acid
NADH: Nicotinamide adenine dinucleotide
NAT: Natural antisense transcript
NCBI: National Center for Biotechnology Information
ncRNA: Non-coding ribonucleic acid
ng: Nanogram
nr: Non-redundant
nt: Nucleotide
ORF: Open reading frame
PEG: Polyethylene glycol
qRT-PCR: Quantitative reverse transcription polymerase chain reaction
Q-score: Quality score
R1: Read 1
R2: Read 2
RNA: Ribonucleic acid
RNA-seq: Ribonucleic acid sequencing
rRNA: Ribosomal ribonucleic acid
SE: Standard error
snoRNA: Small nucleolar ribonucleic acid
UCO: Ultra-conserved ortholog
VLCFA: Very long chain fatty acid
μL: Microliter
μM: Micromolar
