# Supplementary material for: De novo transcriptome assembly reveals high transcriptional complexity in Pisum sativum axillary buds and shows rapid changes in expression of diurnally regulated genes
Source: BMC Genomics. 2017 Mar 2;18:221. doi: 10.1186/s12864-017-3577-x (PMC5335751; doi:10.1186/s12864-017-3577-x)
Supplement: Additional file 1: Table S1. — Summary of the statistics for the raw and cleaned MiSeq paired-end reads. Table S2. Read quality statistics for Read 1 and Read 2 of each of the 24 RNA libraries sequenced. Figure S1. Length distribution of the isoforms in the pea axillary bud transcriptome. Table S3. Comparison of the Pisum sativum bud transcriptome isoforms using BLASTN (1E−03) and specifying a minimum hit coverage of 80%. Table S4. BLASTX (1E−100) results comparing single copy pea reference sequences against the pea axillary bud transcriptome. Figure S12. Length distribution of the long non-coding RNA in the pea axillary bud transcriptome. Table S8. Summary of the number of sequences with similarity to a pea repetitive DNA sequence [23] using BLAST (1E−10). Figure S13. The carbon fixation in photosynthetic organisms KEGG pathway with enzymes highlighted that were identified in the pea axillary bud transcriptome. Table S16. Timetable of sample harvest for the differential expression analysis. Table S17. List of 37 transcripts identified as DE in a pairwise comparison between time window 2 and time window 3. Table S18. List of 124 transcripts identified as DE in a pairwise comparison between time window 1 and time window 3. Table S19. List of 31 DE transcripts annotated with a long non-coding RNA. Table S20. List of DE transcripts that are bound or upregulated by PRR5 [34]. Table S21. List of DE transcripts that have previously been identified as circadian clock-associated and/or light/diurnally-regulated [58–71]. Table S22. List of 29 DE transcripts identified as diurnally regulated in Blasing et al. [35]. Table S23. List of online resources used in this article and their sources. Table S24. List of primers used for qRT-PCR analysis. (PDF 253 kb) [file 12864_2017_3577_MOESM1_ESM.pdf]

Table S1: Summary of the statistics for the raw and cleaned MiSeq paired-end reads

	**Raw**	**Cleaned**
Total # paired-end reads	101,707,414	90,049,877
Ave. read length (bp)	196	122
Ave. Q20 %	29.8 ± 0.52	33.8 ± 0.22
Ave. N%	1.42 ± 0.58	0.59 ± 0.29
Ave. GC %	43.8 ± 0.38	42.2 ± 0.39

Table S2: Read quality statistics for Read 1 and Read 2 of each of the 24 RNA libraries sequenced

					Before cleaning			After cleaning		
Sample	Treatment	Treatment length (hours)	Harvest time window	Read	Q20 %	N %	GC %	Q20 %	N %	GC %
1	Mock	1	1 (13:30-14:10)	1	27.03	3.24E-02	42	33.68	2.58E-03	41
				2	32.23	1.34E-02	41	34.41	2.33E-03	40
2	*rac*-GR24	1	1 (13:30-14:10)	1	29.14	7.94E-02	40	33.09	4.33E-03	38
				2	34.7	5.61E-02	39	35.54	1.21E-02	38
3	Mock	2	1 (13:30-14:10)	1	28.92	1.10E-01	42	33	4.12E-03	41
				2	34.92	5.47E-02	40	35.96	1.24E-02	39
4	*rac*-GR24	2	1 (13:30-14:10)	1	27.04	8.57E-02	43	33.15	8.82E-03	41
				2	32.25	1.67E-02	40	33.86	1.29E-02	39
5	Mock	4	1 (13:30-14:10)	1	25.17	1.80E-01	46	34.07	5.12E-03	44
				2	32.17	1.57E-02	46	34.07	1.42E-02	46
6	*rac*-GR24	4	1 (13:30-14:10)	1	26.88	8.84E-02	45	33.67	2.41E-03	43
				2	31.81	1.32E-02	45	33.89	2.25E-03	44
7	Mock	6	1 (13:30-14:10)	1	26.54	1.49E-01	46	32.43	8.45E-03	44
				2	31.81	1.75E-02	46	33.47	1.27E-02	45
8	*rac*-GR24	6	1 (13:30-14:10)	1	25.45	6.84E-01	45	32.22	3.56E-03	42
				2	23.7	14.9	45	29.25	6.18	43
9	Mock	1	2 (14:10-14:50)	1	26.71	4.15E-02	43	33.05	2.83E-04	41
				2	32.27	1.74E-02	41	33.62	1.00E-02	40
10	*rac*-GR24	1	2 (14:10-14:50)	1	28.69	2.79E-02	48	32.8	3.85E-03	47
				2	34.61	5.67E-02	50	35.73	1.38E-02	48
11	Mock	2	2 (14:10-14:50)	1	25.58	6.08E-01	44	32.31	3.71E-03	41
				2	24.26	14.75	41	29.63	6.34	40
12	*rac*-GR24	2	2 (14:10-14:50)	1	25.43	3.58E-02	44	34.34	4.90E-03	41
				2	33.61	1.54E-02	42	35.42	1.51E-02	40
13	Mock	4	2 (14:10-14:50)	1	33.32	1.02E-01	41	34.59	6.81E-03	41
				2	35.08	1.05E-01	41	35.79	1.17E-02	39
14	*rac*-GR24	4	2 (14:10-14:50)	1	26.9	1.26	45	33.83	2.72E-03	44
				2	30.91	1.29E-02	45	34.17	2.41E-03	43
15	Mock	6	2 (14:10-14:50)	1	25.36	4.89E-02	44	34.14	4.84E-03	41
				2	31.7	1.53E-02	41	33.95	1.35E-02	40
16	*rac*-GR24	6	2 (14:10-14:50)	1	33.24	8.29E-02	42	34.52	6.41E-03	41
				2	35.39	1.06E-01	41	35.98	1.21E-02	40
17	Mock	1	3 (15:50-16:20)	1	28.49	3.27E-02	49	32.67	4.08E-03	47
				2	33.9	5.52E-02	51	35.13	1.28E-02	50
18	*rac*-GR24	1	3 (15:50-16:20)	1	25.6	4.13E-02	44	34.51	5.04E-03	42
				2	32.61	1.51E-02	42	34.8	1.41E-02	41

19	Mock	2	3 (15:50-16:20)	1	27.3	9.82E-02	43	33.83	2.85E-03	42
				2	31.37	1.28E-02	43	33.85	2.03E-03	42
20	*rac*-GR24	2	3 (15:50-16:20)	1	25.96	6.64E-01	44	32.59	4.09E-03	41
				2	25.78	14.8	42	30.48	9.32	40
21	Mock	4	3 (15:50-16:20)	1	29.16	2.16	46	33.37	8.87E-03	45
				2	23.53	13.5	46	31.03	6.18	44
22	*rac*-GR24	4	3 (15:50-16:20)	1	33.14	8.23E-01	46	34.89	7.09E-03	46
				2	34.27	1.01E-01	46	35.77	1.20E-02	45
23	Mock	6	3 (15:50-16:20)	1	33.1	6.72E-01	44	34.76	6.77E-03	43
				2	34.22	9.83E-02	45	35.53	1.02E-02	43
24	*rac*-GR24	6	3 (15:50-16:20)	1	29.33	1.18	43	34.14	1.28E-02	41
				2	31.18	1.65E-02	43	34.73	1.22E-02	40

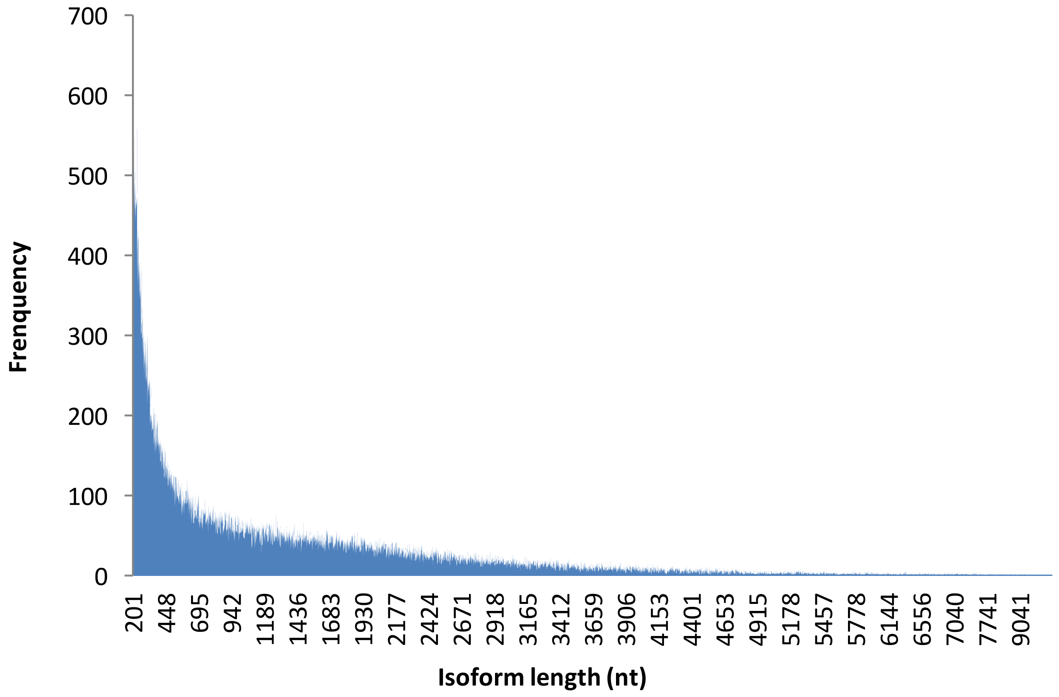

Figure S1: Length distribution of the isoforms in the pea axillary bud transcriptome

Table S3: Comparison of the *Pisum sativum* bud transcriptome isoforms using BLASTN ($1E^{-03}$) and specifying a minimum hit coverage of 80%.

**Type of match**	**# matches**
Self	194,020
Different isoform within the same transcript	56,077
Different transcript	6
**Total**	**250,103**

Table S4: BLASTX ($1E^{-100}$) results comparing single copy pea reference sequences against the pea axillary bud transcriptome

Pea sequence	Sequence type	Pea sequence length (bp)	Transcriptome match	Transcriptome match length (bp)	Alignment length (bp)	Pea sequence coverage	Positives	Gaps
PsApxI	mRNA	1054	comp92406_c0_seq1	1674	1034	98.0%	1033	1
PsBRC1	CDS	1367	comp78442_c0_seq1	2079	1170	85.3%	1166	0
PsEXGT1	mRNA	1125	comp88216_c0_seq5	1248	1124	99.5%	1119	0
			comp88216_c0_seq11	1080	821	72.9%	820	0
			comp88216_c0_seq8	1170	627	55.7%	627	0
			comp88216_c0_seq13	1580	648	56.6%	637	0
			comp88216_c0_seq1	1256	648	56.6%	637	0
			comp88216_c0_seq14	1412	345	30.0%	338	0
			comp88216_c0_seq7	1088	345	30.0%	338	0
PsFed-1	CDS	1995	comp55599_c1_seq1	1079	900	45.1%	900	0
			comp351273_c0_seq1	1058	527	25.5%	509	9
PsHMG-1	mRNA	807	comp81333_c0_seq1	1208	798	97.9%	790	0
PsKO1	mRNA	1797	comp71699_c1_seq1	1791	1754	97.6%	1754	0
			comp71699_c1_seq4	1977	1627	90.5%	1627	0
			comp71699_c1_seq3	2076	1164	64.7%	1163	0
			comp71699_c1_seq2	1890	1164	64.7%	1163	0
PsPCNA	mRNA	948	comp93178_c0_seq1	1095	926	96.8%	918	5
PsPETE	Genomic	1505	comp92360_c0_seq1	1084	1044	69.0%	1038	2
PsRMS4	CDS	2127	comp97254_c0_seq1	2895	2127	99.8%	2123	0
PsRMS5	CDS	3880	comp74555_c0_seq1	1085	788	20.0%	776	6
			comp27382_c0_seq1	423	291	7.4%	289	0

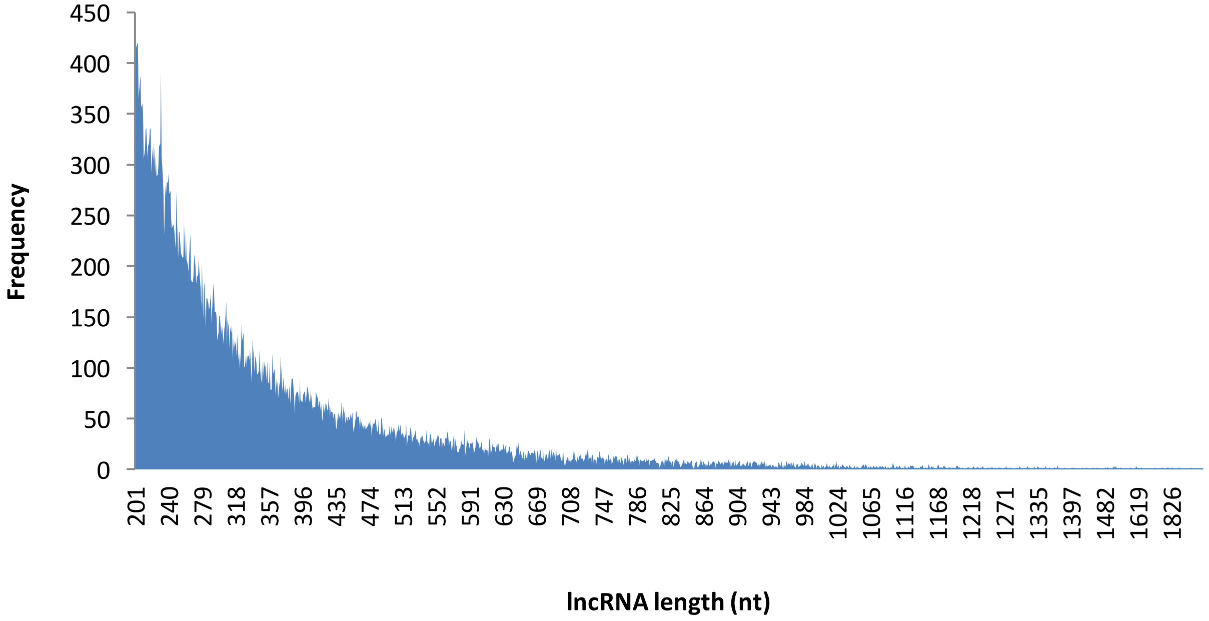

Figure S12: Length distribution of the long non-coding RNA in the pea axillary bud transcriptome

Table S8: Summary of the number of sequences with similarity to a pea repetitive DNA sequence [23] using BLAST ($1E^{-10}$)

Abbrev.	Type of repeat	Transcriptome All Isoforms	Transcriptome Longest Isoform	lncRNA
RLGTT	LTR/gypsy/Tat	267	118	48
RLGOG	LTR/gypsy/Ogre	4,307	1,625	2,094
RLGCH	LTR/gypsy/chromo	317	127	138
RLGAT	LTR/gypsy/Athila	936	336	259
RLCTK	LTR/copia/Tork	167	46	23
RLCTR	LTR/copia/TAR	76	29	10
RLCMX	LTR/copia/Maximus	1,247	439	364
RLCIV	LTR/copia/Ivana	512	158	42
RLCAN	LTR/copia/Angela	87	22	41
RLCA2	LTR/copia/AleII	219	31	16
RLCA1	LTR/copia/AleI	8	3	1
RLXXX	LTR/unclassified	387	89	117
TRIMx	LTR/TRIM	97	46	50
DHHxx	Helitron	10	6	0
DTMxx	DNA/MULE	230	78	55
DTAxx	DNA/hAT	7	3	0
DTCxx	DNA/CACTA	395	40	33
SAT09	Satellite/PST_TR9	1	1	1
SAT07	Satellite/PST_TR7	0	0	0
SAT03	Satellite/PST_TR3	1	1	1
SAT27	Satellite/PST_TR27	3	1	0
SAT25	Satellite/PST_TR25	2	1	0
SAT11	Satellite/PST_TR11_TR19	62	15	58
SAT01	Satellite/PST_TR1	24	3	16
SAT06	Satellite/PS_TR6	1	1	1
SAT0B	Satellite/PisTR-B	2	2	2
SATxx	Satellite/other	20	4	13
RDN05	rDNA/5S	1	1	0
RDN45	rDNA/45S	6	6	2
CONTM	contam.	1	1	0
PLAST	plastid	24	13	0
XXXXX	unclassified repeat	2,617	954	789
	**Total repeats**	**12,034**	**4,200**	**4,174**
	**Total in set**	**194,067**	**81,773**	**47,322**

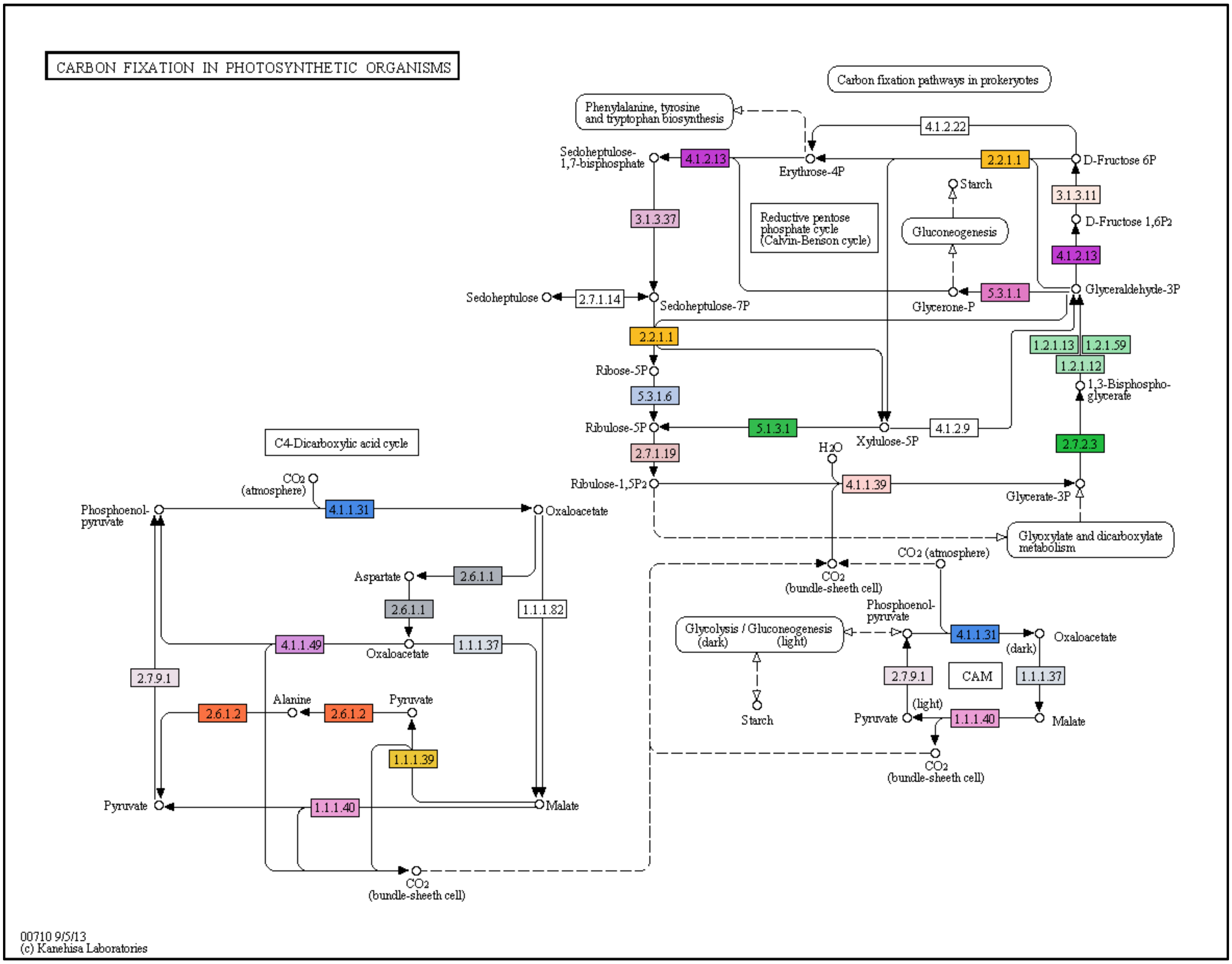

Figure S13: The carbon fixation in photosynthetic organisms KEGG pathway with enzymes highlighted that were identified in the pea axillary bud transcriptome.

Table S16: Timetable of sample harvest for the differential expression analysis

**Time window**	**1**	**2**	**3**
**Time**	13:30 - 14:10	14:10 - 14:50	15:50 - 16:20
**Samples harvested**	1, 3, 5, 7	9, 11, 13, 15	17, 19, 21, 23

Table S17: List of 37 transcripts identified as DE in a pairwise comparison between time window 2 and time window 3

Transcript	Fold change	FDR
comp55670_c0	29.99273454	6.71E-05
comp38965_c0	67.96124778	6.74E-05
comp91844_c6	21.89150914	6.74E-05
comp35107_c1	12.87711767	0.000615261
comp91844_c0	191.4521392	0.000615261
comp80518_c0	155.7358145	0.000615261
comp92923_c0	37.90478548	0.000615261
comp81803_c0	2.937871057	0.001049039
comp388637_c0	66.33635356	0.001480698
comp86806_c0	84.25789332	0.001568536
comp51650_c0	86.01018718	0.001568536
comp72075_c0	2.345996512	0.001756021
comp55670_c1	47.76202731	0.001858386
comp90592_c12	12.9588119	0.003091317
comp91844_c4	30.36488816	0.00500408
comp91844_c5	92.66608899	0.008037182
comp79487_c0	0.105749722	0.012025699
comp55670_c2	16.62011456	0.012465091
comp82517_c0	4.349992898	0.012465091
comp35755_c0	5.244088628	0.020082174
comp55149_c0	0.503804682	0.02193618
comp92265_c0	9.22923673	0.02193618
comp89084_c0	29.43663111	0.02193618
comp48055_c0	28.22927049	0.023531557
comp83707_c0	2.034885925	0.023921647
comp89415_c1	2.098533173	0.026265933
comp91844_c1	37.55825232	0.028886441
comp62497_c0	13.89751364	0.031060459
comp87035_c5	0.522368803	0.032163598
comp75928_c0	85.09750094	0.036731105
comp82468_c1	6.646879517	0.036936271
comp35877_c1	8.558968348	0.036936271
comp86920_c1	0.42039615	0.041893328
comp91844_c3	85.10279393	0.041893328
comp255391_c0	0.122154402	0.041893328
comp87071_c2	5.591675309	0.041893328
comp35285_c0	2.18272596	0.046608407

Table S18: List of 124 transcripts identified as DE in a pairwise comparison between time window 1 and time window 3

Transcript	Fold Change	FDR
comp35107_c1	41.62059352	1.63E-08
comp92265_c0	44.16514369	5.14E-07
comp38965_c0	124.5553127	1.69E-06
comp86806_c0	351.5948264	8.15E-05
comp80157_c2	4.875100562	9.92E-05
comp89617_c0	0.283276641	9.92E-05
comp78391_c2	3.078677156	0.000177796
comp35693_c0	0.254851951	0.000540388
comp72468_c0	0.234277618	0.000690412
comp81803_c0	2.971293206	0.000773246
comp89415_c1	2.536580022	0.000773468
comp42921_c0	2.44513865	0.000837202
comp388637_c0	66.33635356	0.001104366
comp92923_c0	32.52828075	0.00121689
comp94434_c0	2.402020453	0.001359336
comp94479_c0	8.677547285	0.001481186
comp51650_c0	72.39438954	0.001671554
comp35102_c0	2.609360842	0.001671554
comp72075_c0	2.271907111	0.002768533
comp87227_c2	0.281042559	0.003059639
comp93451_c0	0.490816238	0.003076272
comp75928_c0	296.4334779	0.003076272
comp55051_c0	0.458834444	0.003522094
comp89084_c0	46.10386084	0.003547779
comp77929_c0	2.476602443	0.003547779
comp92807_c0	4.369939288	0.004278948
comp54867_c0	0.158205609	0.004464485
comp86920_c1	0.384591552	0.005495493
comp81416_c0	0.281721148	0.005495493
comp35297_c0	0.307749793	0.005495493
comp90925_c6	0.386170581	0.005578998
comp71289_c1	2.450371111	0.005848849
comp35767_c0	50.88281029	0.006125543
comp91531_c2	0.488835605	0.006125543
comp35134_c1	5.078354074	0.006331317
comp91844_c6	9.588393283	0.006331317
comp62653_c1	0.264478854	0.006950003
comp93714_c0	2.345100401	0.008421684
comp75525_c2	3.4475625	0.008518746

comp69006_c0	0.193777858	0.008660182
comp101029_c0	8.342688272	0.009146392
comp55866_c0	2.743141967	0.009146392
comp90592_c12	9.745642207	0.009146392
comp96310_c0	5.177483256	0.010631298
comp64053_c1	2.383494979	0.011046193
comp77793_c3	3.703659127	0.011591791
comp88866_c1	18.25062458	0.011591791
comp90918_c1	0.238067911	0.011591791
comp92821_c0	0.234629843	0.011591791
comp83232_c1	0.418244114	0.01212127
comp35188_c0	2.512101169	0.01212127
comp84301_c1	0.528306176	0.013802408
comp85451_c1	4.054877957	0.013802408
comp93257_c0	0.435928722	0.013802408
comp34724_c0	0.267386195	0.013802408
comp96168_c0	0.387384733	0.013802408
comp86806_c1	35.31901296	0.014355245
comp81954_c0	0.144236576	0.014699988
comp77793_c2	3.300349104	0.015079458
comp62497_c0	13.79001673	0.015675878
comp71932_c0	2.191711532	0.015675878
comp35867_c0	0.463667913	0.016037764
comp103811_c0	0.438422109	0.016086194
comp78122_c1	0.510227368	0.019400733
comp77973_c0	3.614087566	0.019400733
comp147611_c0	0.174430724	0.020012179
comp71348_c1	6.096575179	0.020012179
comp55670_c0	8.477802142	0.020012179
comp87716_c1	0.424807026	0.020012179
comp31797_c1	0.036780193	0.020012179
comp89772_c0	0.489748035	0.021058981
comp592517_c0	0.184439745	0.021058981
comp88228_c0	11.34001519	0.021275559
comp93952_c0	2.215977928	0.02142318
comp35285_c0	2.199379674	0.021539194
comp84585_c0	0.54072815	0.021565982
comp99868_c0	3.556355483	0.021565982
comp79509_c0	0.244724285	0.02193375
comp79487_c0	0.124821608	0.022851406
comp84080_c0	0.525602531	0.023148154
comp70446_c0	0.485318874	0.023631247

comp84940_c2	24.32895611	0.023829678
comp91837_c4	24.32444728	0.023829678
comp77858_c0	2.726496511	0.023829678
comp89398_c0	0.215875969	0.024801449
comp82323_c0	2.64569673	0.02779823
comp414989_c0	24.37367646	0.028369781
comp90486_c1	0.366662457	0.030502355
comp73339_c0	3.275138883	0.030744869
comp38609_c0	0.043311641	0.030744869
comp66814_c0	0.04334664	0.030744869
comp79848_c1	0.039679042	0.031876991
comp85721_c1	0.371170827	0.031986683
comp86930_c0	0.374360704	0.033530463
comp78315_c0	0.502308382	0.033746451
comp117149_c0	0.100488563	0.033746451
comp35337_c0	5.321273778	0.034945224
comp79176_c0	0.209526904	0.036280221
comp34931_c0	0.414122298	0.036280221
comp88021_c0	0.320873657	0.037224073
comp55974_c0	0.366132034	0.038195687
comp378614_c0	0.042646378	0.038246215
comp74630_c0	7.592060618	0.038391387
comp75279_c0	22.35969196	0.039156917
comp87121_c0	0.460622995	0.039277231
comp93445_c0	2.127930473	0.039442018
comp34949_c0	2.858494038	0.04080457
comp83562_c0	0.383569793	0.041149023
comp82340_c1	0.414984732	0.043924934
comp34934_c1	0.200406735	0.043924934
comp89250_c4	0.35787063	0.044566686
comp303244_c0	14.94919677	0.047008271
comp94085_c0	3.157270063	0.048079281
comp95443_c0	1.824167904	0.048481555
comp35195_c0	0.559542992	0.048525017
comp86255_c2	0.357267624	0.048525017
comp36828_c0	10.03561238	0.048525017
comp82539_c0	10.02910353	0.048525017
comp85037_c0	10.02113494	0.048525017
comp70806_c0	2.497715551	0.048525017
comp83593_c0	0.497083606	0.048525017
comp252844_c0	0.047485263	0.048525017
comp94060_c0	5.836095459	0.048677117

comp68925_c0	1.83125968	0.049249639

Table S19: List of 31 DE transcripts annotated with a long non-coding RNA

comp35693_c0
comp72468_c0
comp86920_c1
comp81416_c0
comp91531_c2
comp62653_c1
comp75525_c2
comp101029_c0
comp90918_c1
comp96168_c0
comp62497_c0
comp77973_c0
comp147611_c0
comp71348_c1
comp592517_c0
comp88228_c0
comp84940_c2
comp414989_c0
comp90486_c1
comp38609_c0
comp66814_c0
comp378614_c0
comp74630_c0
comp34949_c0
comp82340_c1
comp94085_c0
comp86255_c2
comp252844_c0
comp48055_c0
comp255391_c0
comp87071_c2

Table S20: List of DE transcripts that are bound or upregulated by PRR5 [34]

Transcript	*Arabidopsis* gene name	*Arabidopsis* gene annotation	AT#	Bound by PRR5?	Upregulated by PRR5?
comp91531_c2	RSH2	RELA-SPOT HOMOLOG 2	AT3G14050	Yes	No
comp89415_c1	JMJD5/ JMJ30	JUMONJI DOMAIN CONTAINING 5 / JUMONJI-C DOMAIN-CONTAINING PROTEIN 30	AT3G20810	Yes	No
comp78122_c1	LNK1/2	NIGHT LIGHT-INDUCIBLE AND CLOCK-REGULATED 2	AT3G54500	Yes	Yes
comp93952_c0	RPL23aB	60s ribosomal protein L23a-2	AT3G55280	Yes	No
comp35195_c0	TIR1	TRANSPORT INHIBITOR RESPONSE 1	AT3G62980	Yes	No
comp71932_c0	QCR7	Cytochrome b-c1 complex subunit 7-1	AT4G32470	Yes	No
comp83562_c0	BBX19	B-BOX DOMAIN PROTEIN 19	AT4G38960	No	Yes
comp35134_c1	-	Histone h2b	AT5G02570	Yes	No
comp90486_c1	PRR5	PSEUDO-RESPONSE REGULATOR 5	AT5G24470	Yes	Yes
comp84080_c0	-	P-loop containing nucleoside triphosphate hydrolases superfamily protein	AT5G35970	No	Yes

Table S21: List of DE transcripts that have previously been identified as circadian clock-associated and/or light/diurnally-regulated

Transcript	*Arabidopsis* gene name	*Arabidopsis* gene annotation	AT#	Supporting references
comp42921_c0	FKF1	FLAVIN-BINDING, KELCH REPEAT, F BOX 1	AT1G68050	[58]
comp70806_c0	BBX21	B-BOX DOMAIN PROTEIN 21	AT1G75540	[59]
comp72075_c0	CRY2	CRYPTOCHROME 2	AT1G04400	[60, 61]
comp78122_c1	LNK1/2	NIGHT LIGHT-INDUCIBLE AND CLOCK-REGULATED 2	AT3G54500	[62]
comp83562_c0	BBX19	B-BOX DOMAIN PROTEIN 19	AT4G38960	[63]
comp84080_c0	-	P-loop containing nucleoside triphosphate hydrolases superfamily protein	AT5G35970	[64]
comp84301_c1	TIC55	TRANSLOCON AT THE INNER ENVELOPE MEMBRANE OF CHLOROPLASTS 55	AT2G24820	[65]
comp84585_c0	PHOT2	PHOTOTROPIN 2	AT5G58140	[66]
comp87227_c2	EPR1	EARLY PHYTOCHROME RESPONSIVE 1	AT1G18330	[67]
comp89415_c1	JMJD5/ JMJ30	JUMONJI DOMAIN CONTAINING 5/JUMONJI-C DOMAIN-CONTAINING PROTEIN 30	AT3G20810	[68]
comp89617_c0	CRY3	CRYPTOCHROME 3	AT5G24850	[69]
comp90486_c1	PRR5	PSEUDO-RESPONSE REGULATOR 5	AT5G24470	[70]
comp91531_c2	RSH2	RELA-SPOT HOMOLOG 2	AT3G14050	[71]

[58] Song YH, Smith RW, To BJ, Millar AJ, Imaizumi T. FKF1 conveys timing information for CONSTANS stabilization in photoperiodic flowering. Science. 2012;336(6084):1045-1049.

[59] Xu D, Li J, Gangappa SN, Hettiarachchi C, Lin F, Andersson MX et al. Convergence of light and ABA signaling on the *ABI5* promoter. PLoS Genet. 2014;10(2):e1004197.

[60] Lin CT, Yang HY, Guo HW, Mockler T, Chen J, Cashmore AR. Enhancement of blue-light sensitivity of *Arabidopsis* seedlings by a blue light receptor cryptochrome 2. Proc Natl Acad Sci U S A. 1998;95(5):2686-2690.

[61] Toth R, Kevei E, Hall A, Millar AJ, Nagy F, Kozma-Bognar L. Circadian clock-regulated expression of phytochrome and cryptochrome genes in *Arabidopsis*. Plant Physiol. 2001;127(4):1607-1616.

[62] Rugnone ML, Soverna AF, Sanchez SE, Schlaen RG, Hernando CE, Seymour DK et al. *LNK* genes integrate light and clock signaling networks at the core of the *Arabidopsis* oscillator. Proc Natl Acad Sci U S A. 2013;110(29):12120-12125.

[63] Li G, Siddiqui H, Teng YB, Lin RC, Wan XY, Li JG et al. Coordinated transcriptional regulation underlying the circadian clock in *Arabidopsis*. Nat Cell Biol. 2011;13(5):616-U275.

[64] Kleine T, Kindgren P, Benedict C, Hendrickson L, Strand A. Genome-wide gene expression analysis reveals a critical role for CRYPTOCHROME1 in the response of *Arabidopsis* to high irradiance. Plant Physiol. 2007;144(3):1391-1406.

[65] Boij P, Patel R, Garcia C, Jarvis P, Aronsson H. *In vivo* Studies on the Roles of Tic55-Related Proteins in Chloroplast Protein Import in *Arabidopsis thaliana*. Mol Plant. 2009;2(6):1397-1409.

[66] Christie JM, Salomon M, Nozue K, Wada M, Briggs WR. LOV (light, oxygen, or voltage) domains of the blue-light photoreceptor phototropin (nph1): Binding sites for the chromophore flavin mononucleotide. Proc Natl Acad Sci U S A. 1999;96(15):8779-8783.

[67] Kuno N, Moller SG, Shinomura T, Xu XM, Chua NH, Furuya M. The novel MYB protein EARLY-PHYTOCHROME-RESPONSIVE1 is a component of a slave circadian oscillator in *Arabidopsis*. Plant Cell. 2003;15(10):2476-2488.

[68] Jones MA, Covington MF, DiTacchio L, Vollmers C, Panda S, Harmer SL. Jumonji domain protein JMJD5 functions in both the plant and human circadian systems. Proc Natl Acad Sci U S A. 2010;107(50):21623-21628.

[69] Pokorny R, Klar T, Hennecke U, Carell T, Batschauer A, Essen LO. Recognition and repair of UV lesions in loop structures of duplex DNA by DASH-type cryptochrome. Proc Natl Acad Sci U S A. 2008;105(52):21023-21027.

[70] Nakamichi N, Kita M, Ito S, Yamashino T, Mizuno T. PSEUDO-RESPONSE REGULATORS, PRR9, PRR7 and PRR5, together play essential roles close to the circadian clock of *Arabidopsis thaliana*. Plant Cell Physiol. 2005;46(5):686-698.

[71] Mizusawa K, Masuda S, Ohta H. Expression profiling of four RelA/SpoT-like proteins, homologues of bacterial stringent factors, in *Arabidopsis thaliana*. Planta. 2008;228(4):553-562.

Table S22: List of 29 DE transcripts identified as diurnally regulated in Blasing *et al.* [35]

comp35188_c0
comp35195_c0
comp42921_c0
comp55866_c0
comp70446_c0
comp70806_c0
comp75525_c2
comp77858_c0
comp77929_c0
comp78122_c1
comp78315_c0
comp79848_c1
comp80157_c2
comp81803_c0
comp83232_c1
comp83562_c0
comp84080_c0
comp84301_c1
comp85451_c1
comp85721_c1
comp89415_c1
comp89617_c0
comp89772_c0
comp91531_c2
comp92821_c0
comp93257_c0
comp93952_c0
comp94434_c0
comp99868_c0

Table S23: List of online resources used in this article and their sources

Databases / transcriptomes	Source
Medicago truncatula CDS	ftp://ftp.jcvi.org/pub/data/m_truncatula/Mt4.0/Annotation/Mt4.0v1/Mt4.0v1_GenesCDSSeq_20130731_1800.fasta
Duarte contigs	http://www.ncbi.nlm.nih.gov/nuccore/GAMJ00000000
Franssen contigs	http://www.ncbi.nlm.nih.gov/nuccore?term=66035%5BBioProject%5D
Kaur contigs	http://www.ccforum.com/content/supplementary/1471-2164-13-104-s1.txt
Kaspa contigs	http://www.ncbi.nlm.nih.gov/nuccore?term=277074%5BbioProject%5D
Parafield contigs	http://www.ncbi.nlm.nih.gov/nuccore?term=277076%5BBioProject%5D
Uni-prot pea proteins	http://www.uniprot.org/uniprot/?query=organism:3888
Swiss-prot pea proteins	http://www.uniprot.org/uniprot/?query=organism:3888+reviewed:yes
Ultra-conserved orthologs	http://cgpdb.ucdavis.edu/cgpdb2/data_analysis/reference_genome_set/Arabidopsis/A_thaliana_ATGC_2006_08_24.protein.COS_ULTRA.fasta

Table S24: List of primers used for qRT-PCR analysis.

Transcript	Forward primer (5' to 3')	Reverse primer (5' to 3')
PsTUBLIN2	AGATGGCTTCAACTTTCATTGG	GCTCTCGGCTTCGGTGA
comp35195_c0	AAAATGGCAGCAAGTCCATC	GGTTGGGGTGGTTATGTTTG
comp35285_c0	TCGATCTGGGAGAAGAATGG	AGCGTGACGAAACTGATTCC
comp55670_c0	GCCGACTTTCGTCTCTGTTC	TAGTTTATCTGGGGCGGATG
comp62497_c0	TGAGGTTTTTGCTTTGATGC	TGCAAAGAATCCCAATTGAT
comp62653_c1	GAATGATGGTTTCGGTTTGAG	TCCCTCCACTCATCTCTCTCTC
comp70446_c0	CGGTGCAGTCTCATAGCAAA	ATCTCCGACATCACGTTTCC
comp72075_c0	CAATTGTTCCCTTGCCAAAT	GGAGGAAATACGATCCAGCA
comp75279_c0	TGGCAAAAGTATGGCTATTGG	AGCATGTAGAGAGGGCTTCG
comp81803_c0	CAAATGGCAATGATGATGGA	ACTCCTCTTGTGGCTGCCTA
comp84301_c1	ATGCCGTGTCCCACTTTATC	TCCATCAGAATGCAAGCAAG
comp86920_c1	GCAATCTGTTTCCACCATCC	GGTTCTAGCTCAGCCAATCG
comp86930_c0	TAATGTTTCTCCCGATCACG	CGAAAACCCGACTAACAACC
comp90486_c1	AACGAGCAAGACCCTAAGCA	CGTTTGAGAATGGGTGAGGT
comp93451_c0	CCACCGCTCGAAATATCAGT	GGGCGTCTCTAGCAACATTC
