# Supplementary material for: De novo transcriptome assembly reveals high transcriptional complexity in Pisum sativum axillary buds and shows rapid changes in expression of diurnally regulated genes
Source: BMC Genomics. 2017 Mar 2;18:221. doi: 10.1186/s12864-017-3577-x (PMC5335751; doi:10.1186/s12864-017-3577-x)

Figure S2: The number of reads aligning to the *PsApxI* mRNA sequence and the alignment between *PsApxI* mRNA and its best hit to the pea bud transcriptome, comp92406_c0_seq1

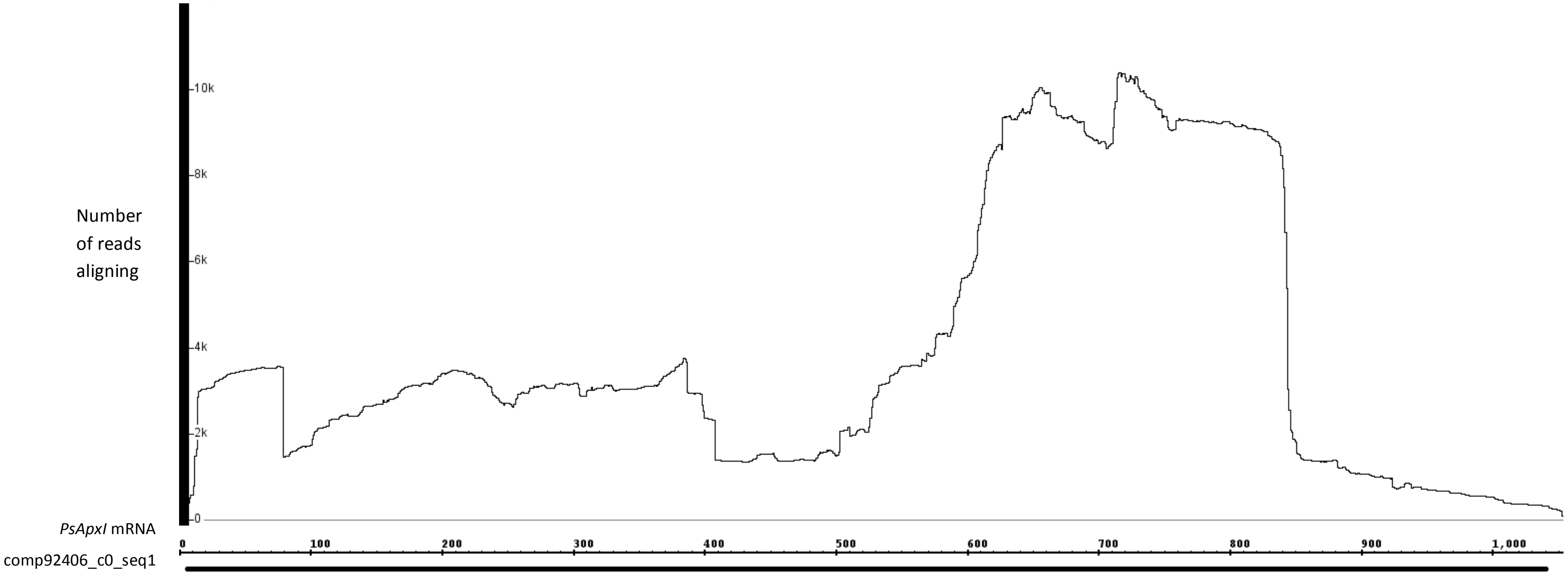

Figure S3: The number of reads aligning to the *PsBRC1* CDS and the alignment between *PsBRC1* CDS and its best hit to the pea bud transcriptome, comp78442_c0_seq1

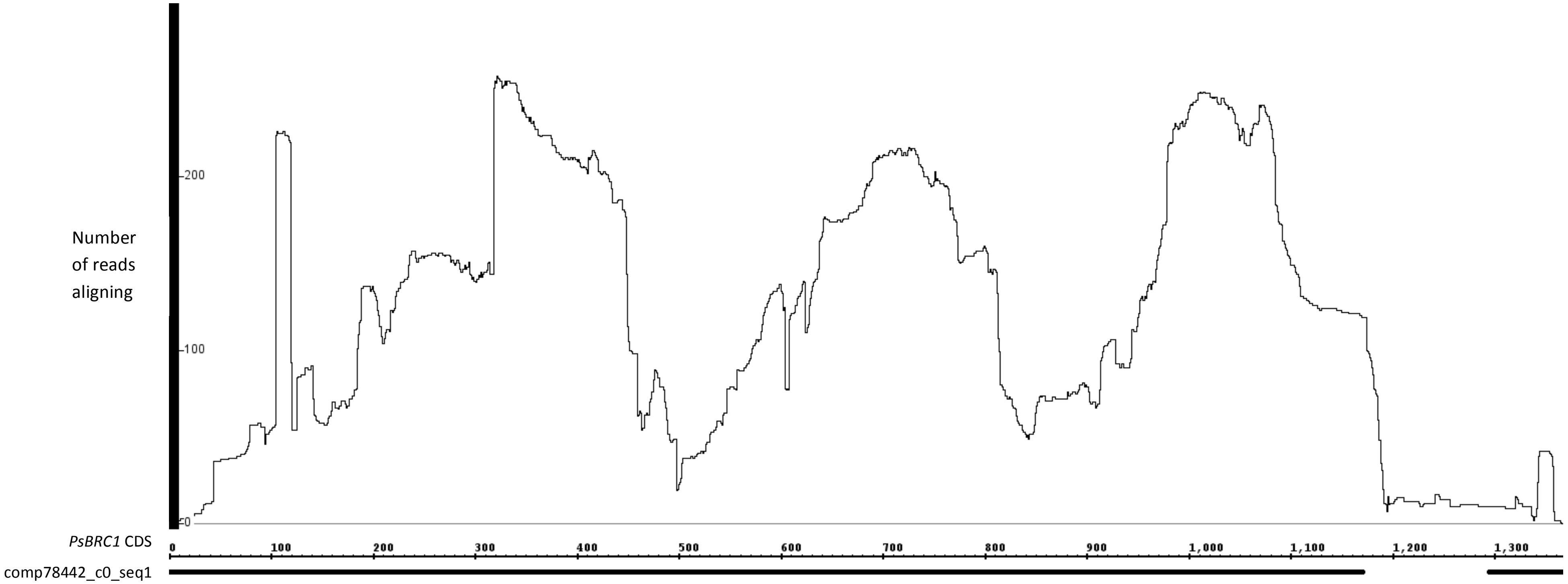

Figure S4: The number of reads aligning to the *PsEXGT1* mRNA and the alignment between *PsEXGT1* mRNA and its best hits to the pea bud transcriptome, comp88216_c0_seq1, 5, 7, 8, 11, 13, 14

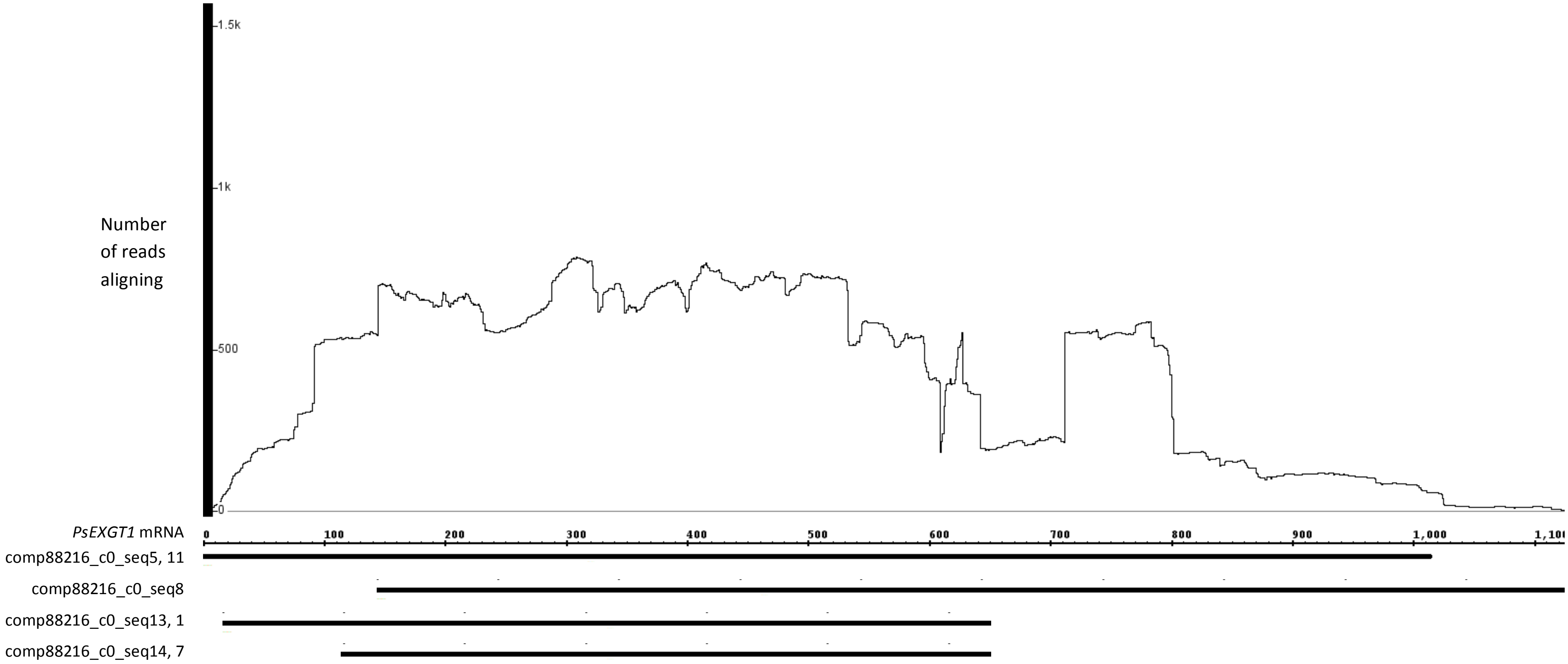

Figure S5: The number of reads aligning to the *PsFed-1* CDS and the alignment between *PsFed-1* CDS and its best hits to the pea bud transcriptome, comp55599_c1_seq1 and comp351273_c0_seq1

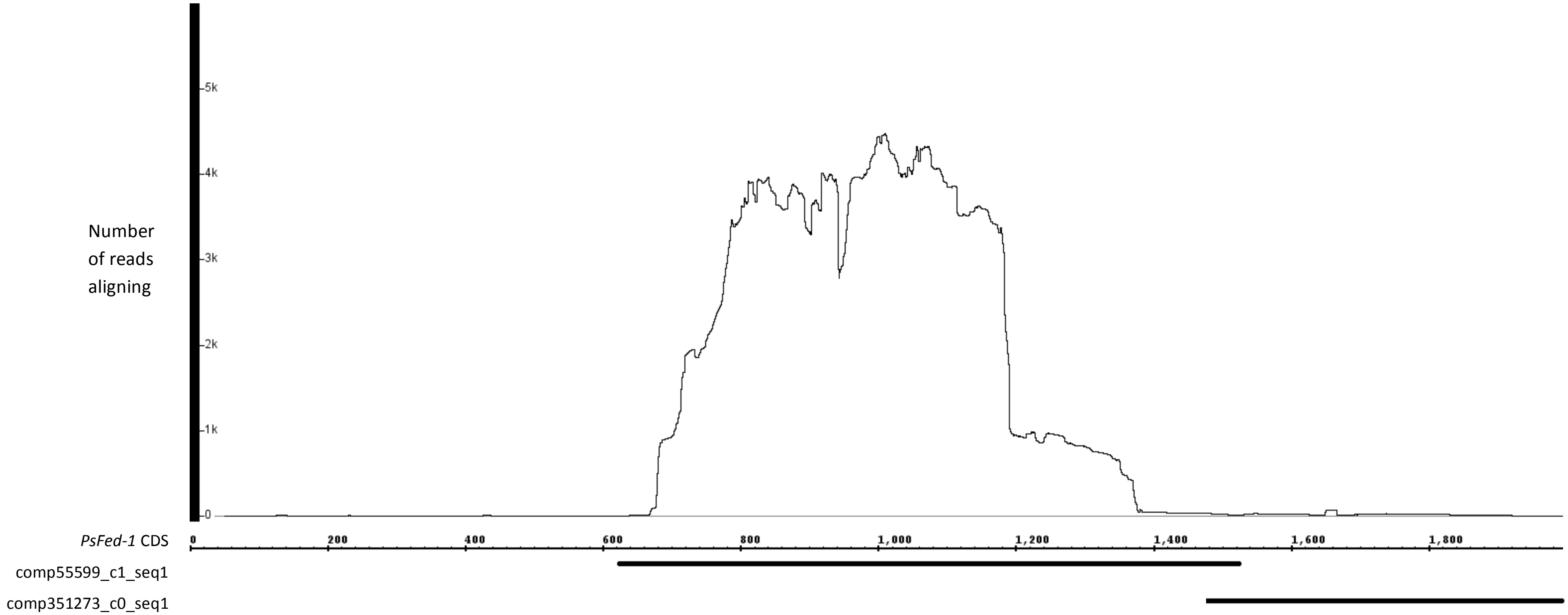

Figure S6: The number of reads aligning to the *PsHMG-1* mRNA and the alignment between *PsHMG-1* mRNA and its best hit to the pea bud transcriptome, comp81333_c0_seq1

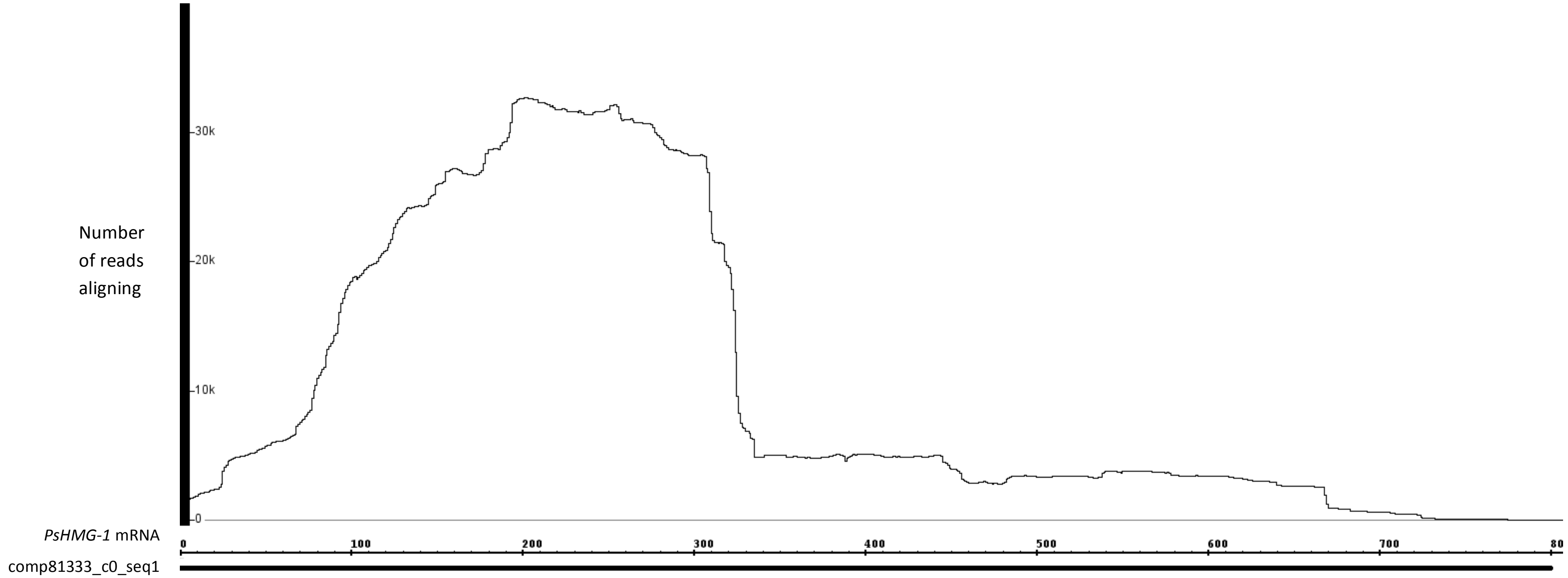

Figure S7: The number of reads aligning to the *PsKO1* mRNA and the alignment between *PsKO1* mRNA and its best hits to the pea bud transcriptome, comp71699_c1_seq1, 2, 3, 4

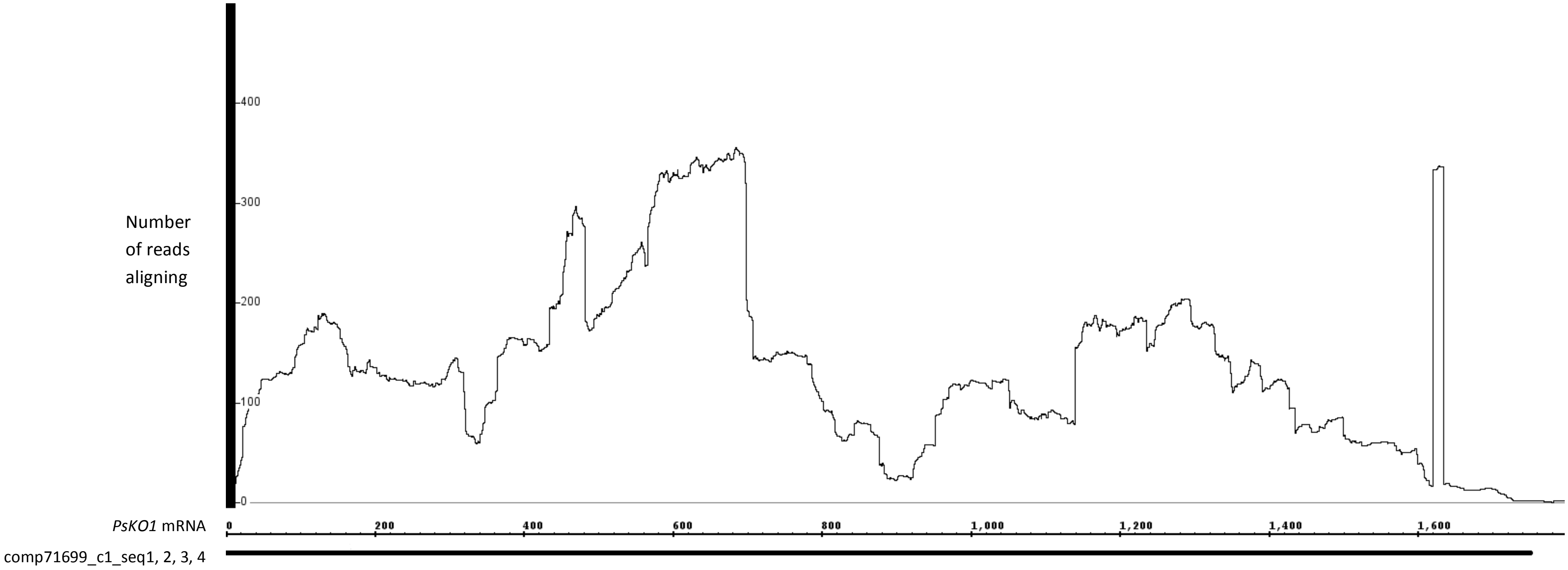

Figure S8: The number of reads aligning to the *PsPCNA* mRNA and the alignment between *PsPCNA* mRNA and its best hit to the pea bud transcriptome, comp93178_c0_seq1

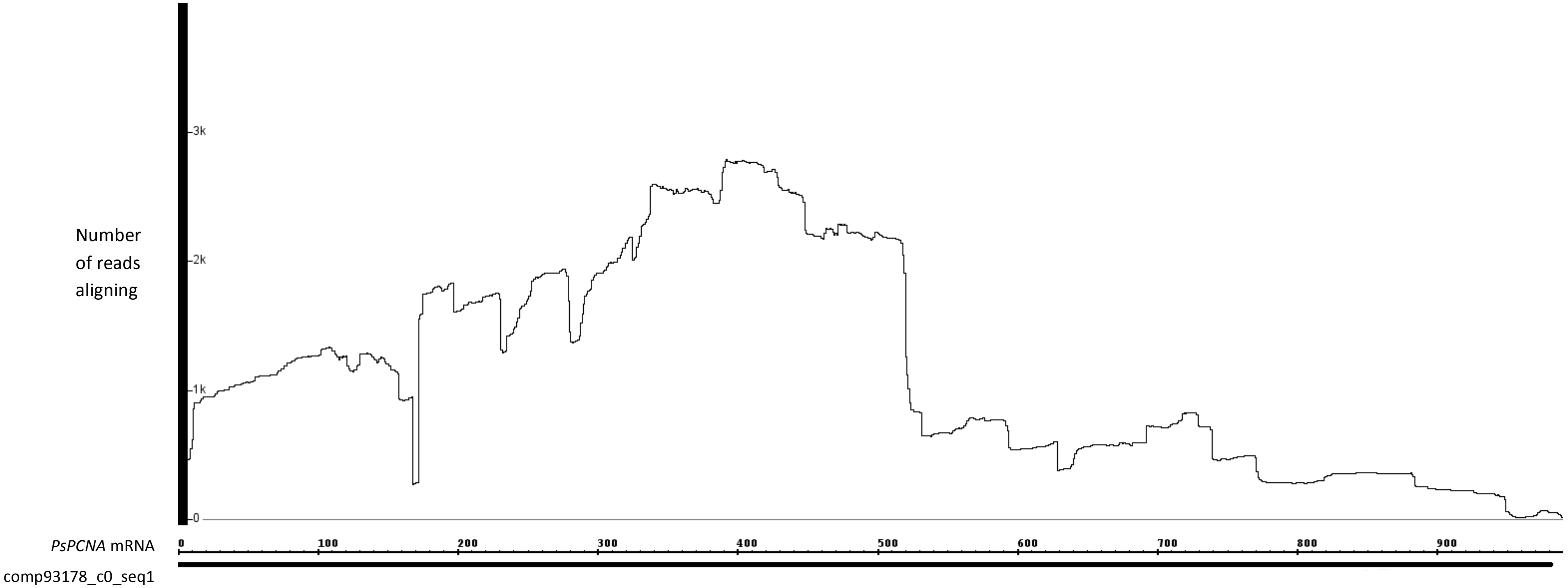

Figure S9: The number of reads aligning to the *PsPETE* gene and the alignment between *PsPETE* gene and its best hit to the pea bud transcriptome, comp92360_c0_seq1

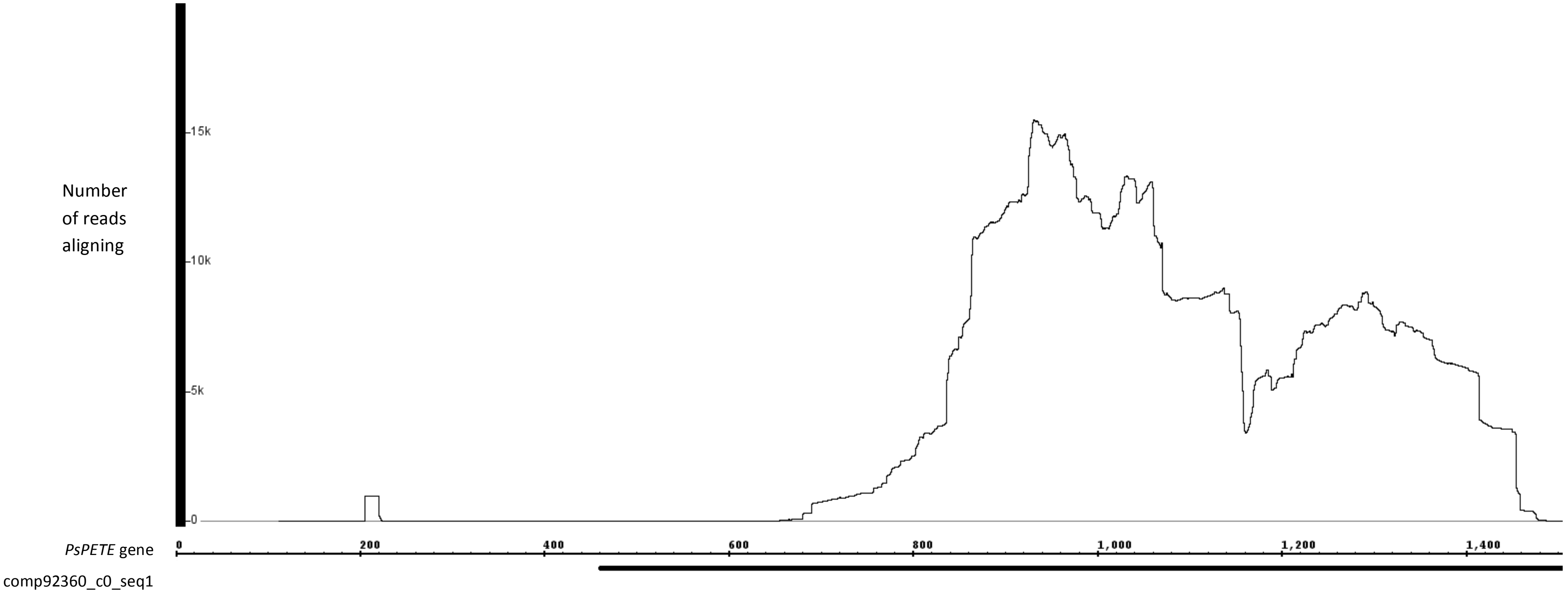

Figure S10: The number of reads aligning to the *PsRMS4* CDS and the alignment between *PsRMS4* CDS and its best hit to the pea bud transcriptome, comp97254_c0_seq1

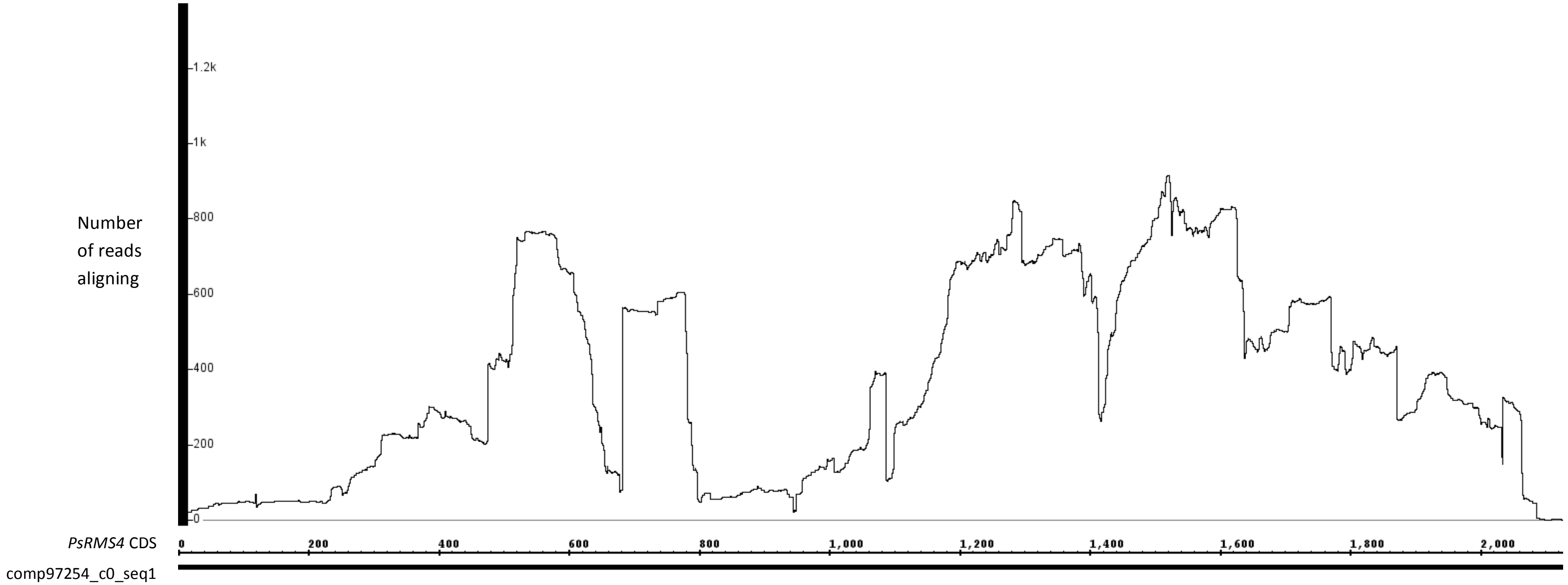

Figure S11: The number of reads aligning to the *PsRMS5* CDS and the alignment between *PsRMS5* CDS and its best hits to the pea bud transcriptome, comp74555_c0_seq1 and comp27382_c0_seq1

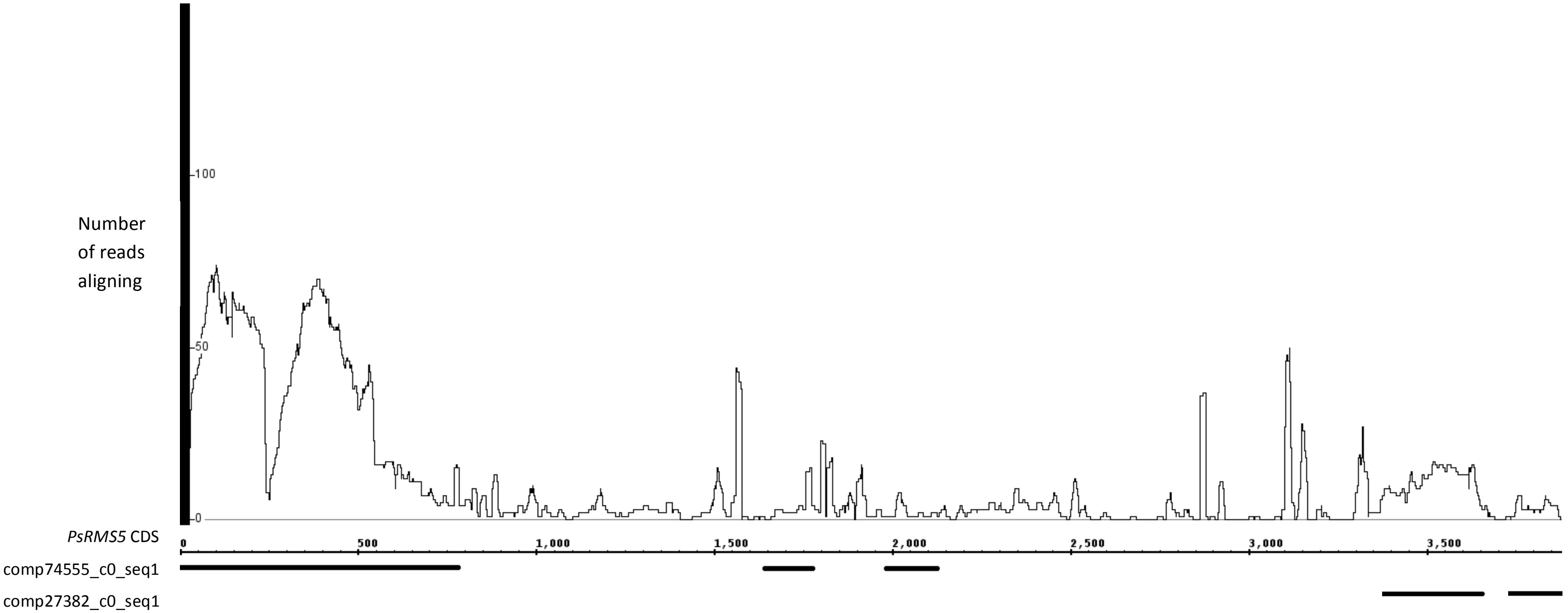

Supplement: Additional file 2: Figure S2. — The number of reads aligning to the PsApxl mRNA sequence and the alignment between PsApxl mRNA and its best hit to the pea bud transcriptome, comp92406_c0_seq1. Figure S3. The number of reads aligning to the PsBRC1 CDS and the alignment between PsBRC1 CDS and its best hit to the pea bud transcriptome, comp78442_c0_seq1. Figure S4. The number of reads aligning to the PsEXGT1 mRNA and the alignment between PsEXGT1 mRNA and its best hits to the pea bud transcriptome, comp88216_c0_seq1, 5, 7, 8, 11, 13, 14. Figure S5. The number of reads aligning to the PsFed-1 CDS and the alignment between PsFed-1 CDS and its best hits to the pea bud transcriptome, comp55599_c1_seq1 and comp351273_c0_seq1. Figure S6. The number of reads aligning to the PsHMG-1 mRNA and the alignment between PsHMG-1 mRNA and its best hit to the pea bud transcriptome, comp81333_c0_seq1. Figure S7 The number of reads aligning to the PsKO1 mRNA and the alignment between PsKO1 mRNA and its best hits to the pea bud transcriptome, comp71699_c1_seq1, 2, 3, 4. Figure S8. The number of reads aligning to the PsPCNA mRNA and the alignment between PsPCNA mRNA and its best hit to the pea bud transcriptome, comp93178_c0_seq1. Figure S9. The number of reads aligning to the PsPETE gene and the alignment between PsPETE gene and its best hit to the pea bud transcriptome, comp92360_c0_seq1. Figure S10. The number of reads aligning to the PsRMS4 CDS and the alignment between PsRMS4 CDS and its best hit to the pea bud transcriptome, comp97254_c0_seq1. Figure S11. The number of reads aligning to the PsRMS5 CDS and the alignment between PsRMS5 CDS and its best hits to the pea bud transcriptome, comp74555_c0_seq1 and comp27382_c0_seq1. (PDF 399 kb) [file 12864_2017_3577_MOESM2_ESM.pdf]
